# Supplementary material for: Variants in ACTC1 underlie distal arthrogryposis accompanied by congenital heart defects
Source: HGG Adv. 2023 Jun 15;4(3):100213. doi: 10.1016/j.xhgg.2023.100213 (PMC10345160; doi:10.1016/j.xhgg.2023.100213)
Supplement: Document S1. Figures S1–S6 and Tables S1 and S2 [file mmc1.pdf]

HGGA, Volume *4*

Supplemental information

Variants in *ACTC1* underlie distal arthrogryposis accompanied by congenital heart defects

Jessica X. Chong, Matthew Carter Childers, Colby T. Marvin, Allison J. Marcello, Hernan Gonorazky, Lili-Naz Hazrati, James J. Dowling, Fatema Al Amrani, Yasemin Alanay, Yolanda Nieto, Miguel Á Marín Gabriel, Arthur S. Aylsworth, Kati J. Buckingham, Kathryn M. Shively, Olivia Sommers, Kailyn Anderson, University of Washington Center for Mendelian Genomics, University of Washington Center for Rare Disease Research, Michael Regnier, and Michael J. Bamshad

Supplemental Materials and Methods

Table of Contents

1. Summary of structural effects of R185W, G199S, and R374S
2. Figure S1. Pedigrees of families with pathogenic variants in *ACTC1* resulting in distal arthrogryposis
3. Figure S2. Histochemistry and immunohistochemistry from muscle biopsy of paravertebral muscles in proband of Family D.
4. Figure S3. Pathogenic variant-associated changes in C_α RMSF of g-actin.
5. Figure S4. Pathogenic variant-associated changes in D-loop – subdomain 2 contacts.
6. Figure S5. Pathogenic variant-associated changes in D-loop secondary structure.
7. Figure S6. Pathogenic variant-associated changes in D-loop solvent accessible surface area.
8. Table S1. *ACTC1* and *ACTA1* missense variants reported as pathogenic in individuals with phenotypes involving cardiac muscle, skeletal muscle, or both. (provided as a separate Excel spreadsheet)
9. Table S2. Reported residue-residue pair interaction frequencies that differed among the WT and mutant simulations. (provided as a separate Excel spreadsheet and in this document)

Summary of structural effects of R185W, G199S, and R374S

R185Y: In the WT simulations, R185 formed transient electrostatic interactions with S16, G17, E74, and D159. Additionally the Y71 side chain formed a large number of atom-atom contacts with R185 by stacking against its guanidino group. Due to these interactions, R185 stabilizes g-actin structure in the vicinity of the ATP pocket and forms a structural linker between all four subdomains. Mutation of R185 to Trp eliminated these stabilizing interactions; instead, W185 was primarily positioned in the cleft between SD2 and SD4 and interacted primarily with other SD4 residues. This resulted in a large-scale reorganization of inter-residue contacts spanning all 4 subdomains and an increase in the SD2-SD4 cleft distance.

G199S: In the WT simulations, G199 adopts backbone dihedral angles that are unfavorable for most amino acids and accommodates a transition from α-helix structure in residues 184-198 to coil structure in residues 199 – 204. Mutation to S199 resulted in a shift in the backbone conformation of residue 199 to angles within the lower left quadrant of Ramachandran space, a decrease in a-helix structure in residues 197-198, and a shift in the structure of the loop spanning residues 199 to 204. This change resulted in few statistically significant changes in inter-residue interactions but did modify the breathing motion between subdomains 2 and 4.

R374S: In the WT simulations, R374 formed transient salt bridges with E363 and E366. Each of these residues are located on the surface of SD1. These salt bridges were lost in the R374S simulations. Simultaneously there was a reorganization of inter-residue interactions in SD1 as well as an increase in residue-residue interactions between an α-helix of SD1 (residues 81-96) and residues in SD2.

Figure S1. Pedigrees of families with pathogenic variants in *ACTC1* resulting in distal arthrogryposis.

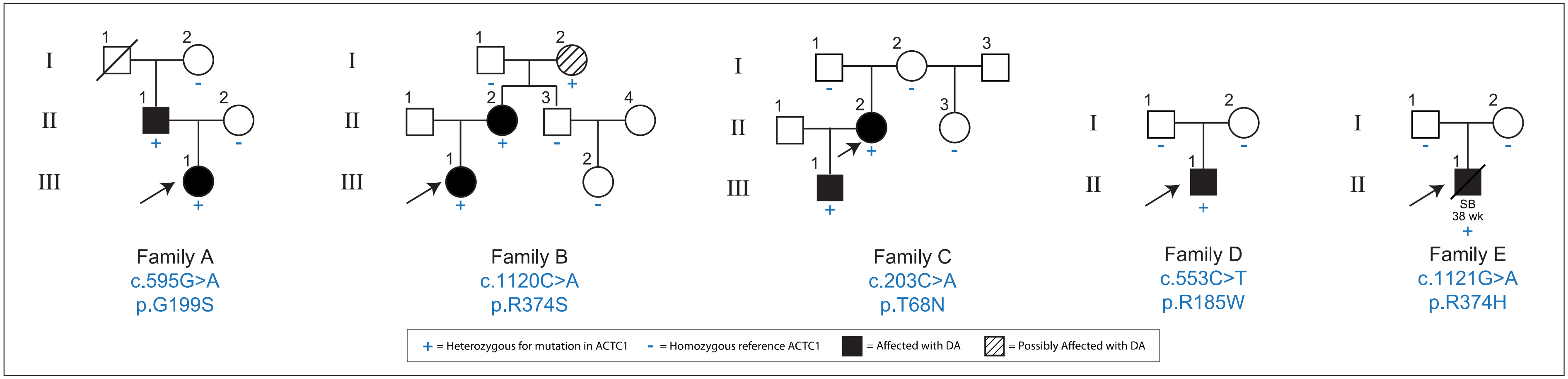

Figure S2. Histochemistry and immunohistochemistry from muscle biopsy of paravertebral muscles in proband of Family D. (A) Scattered areas of round muscle fibers found in H&E [hematoxylin and eosin] staining. (B) ATPase (pH4.2) shows type I fiber predominance that could be seen in paravertebral muscles. (C) No ragged red fibers or rod-like inclusions are seen with modified Gomori trichrome staining however scattered nemaline bodies were found in (D) electronic microscopy.

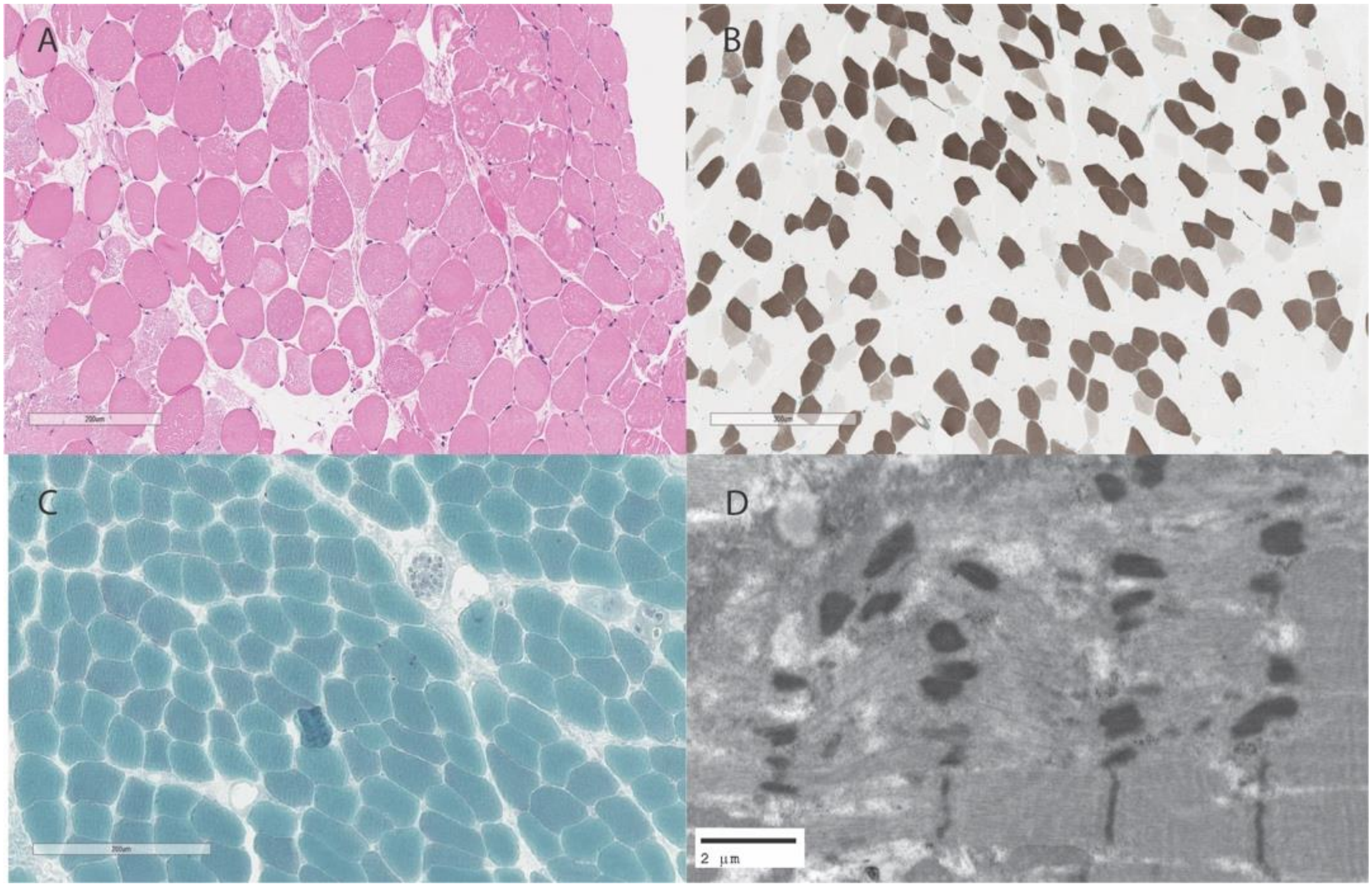

Figure S3. Pathogenic variant-associated changes in C$_\alpha$ RMSF of g-actin. In MD simulations, C$_\alpha$ RMSF measures the root-mean-squared fluctuation of the C$_\alpha$ atoms about their average positions. RMSF values were calculated after alignment of the g-actin trajectories to their average coordinates. Average RMSF values are reported per residue (left column) for the WT (a), T68N (b), R185W (c), G199S (d), and R374S (e)., simulations. Points correspond to the average value and shaded regions denote the standard deviation. Vertical red lines denote mutation sites. The difference in the average RMSF relative to the WT simulation of the T68N (b), R185W (c), G199S (d), and R374S (e) variants are plotted in the right column. Positive values indicate greater RMSF in WT and negative values indicate greater RMSF in the mutants. Vertical red lines denote variant sites. Red stars denote residues with statistically significant ($p < 0.05$) differences in the RMSF between the WT and mutant simulations.

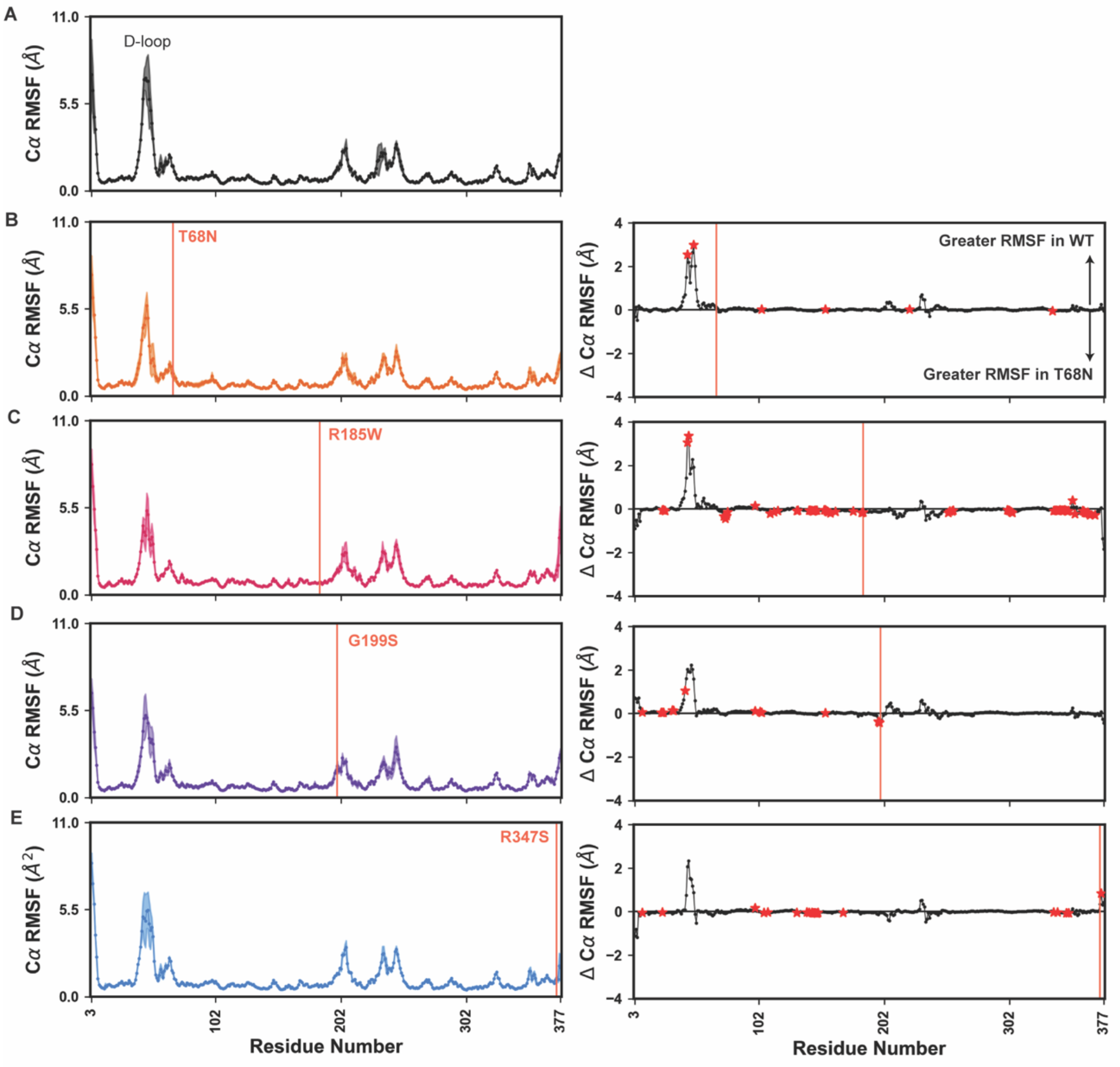

Figure S4. Pathogenic variant-associated changes in D-loop – subdomain 2 contacts. We calculated the average percent simulation time for which select residue-residue interactions were present in the WT (black), T68N (orange), R185W (magenta), G199S (purple), and R374S (blue) simulations. Error bars denote standard deviation. Statistically significant differences between the WT and T68N contact frequencies are denoted (ns: not significant, *: p ≤ 0.05, **: p ≤ 0.01)

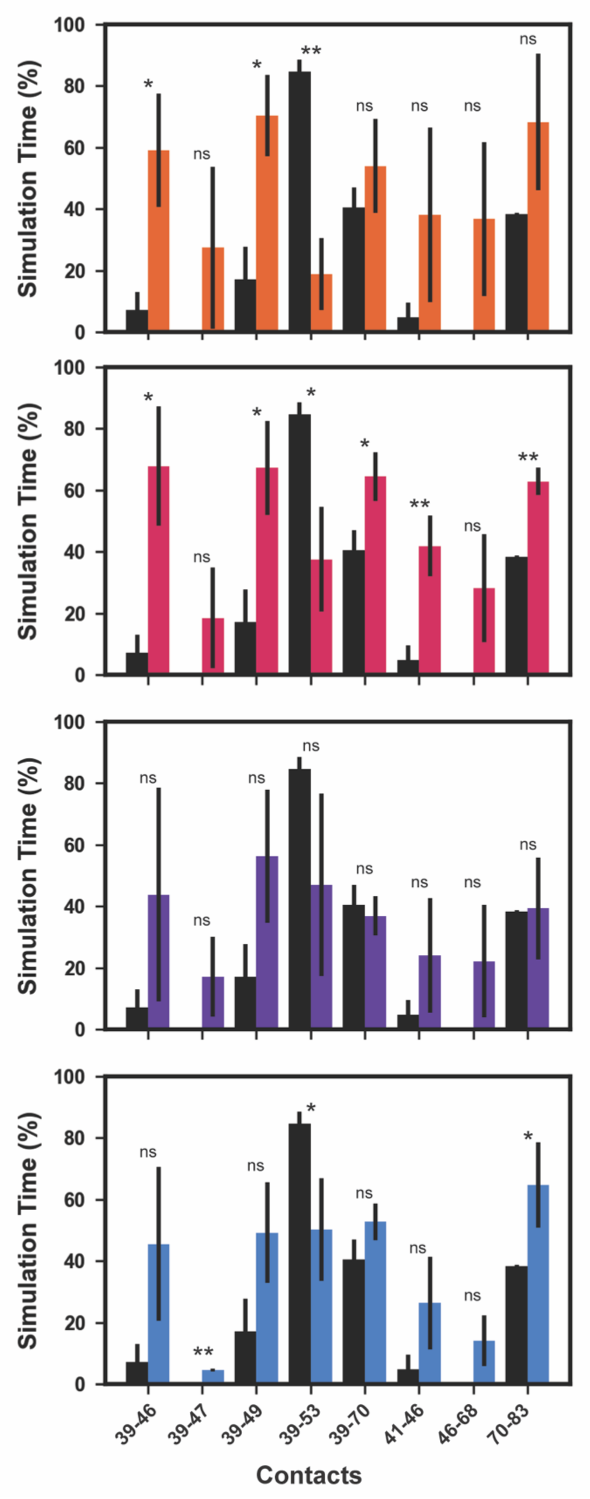

Figure S5. Pathogenic variant-associated changes in D-loop secondary structure. For each residue in the D-loop (# 40 – 56), we calculated the average percent simulation time for which the residue adopted α-helix secondary structure. The α-helix content per residue is shown for T68N (orange), R185W (magenta), G199S (purple), and R374S (blue) versus the WT (grey) simulation. All variants in this study resulted in an increase in α-helix content in the D-loop.

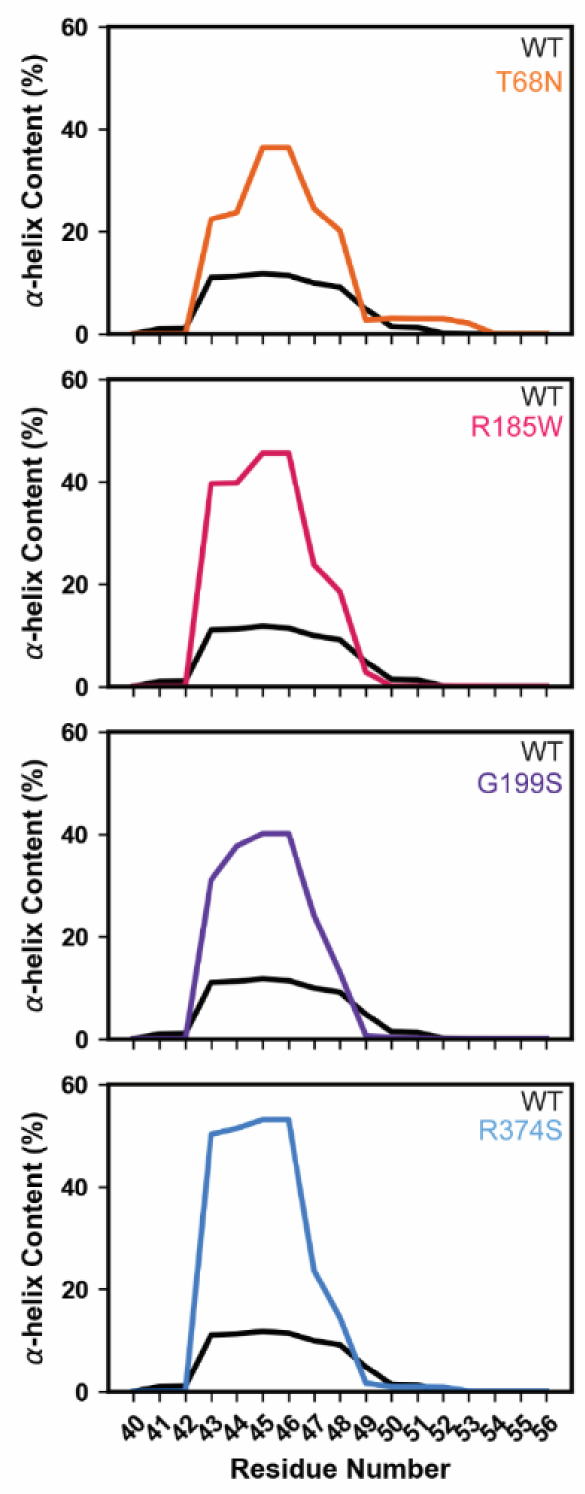

Figure S6. Pathogenic variant-associated changes in D-loop solvent accessible surface area. Histograms of the solvent accessible surface area aggregated over all replicate simulations are plotted for T68N (orange), R185W (magenta), G199S (purple), and R374S (blue) versus the WT (grey) distribution. All variants in this study resulted in a decrease in the SASA of D-loop residues.

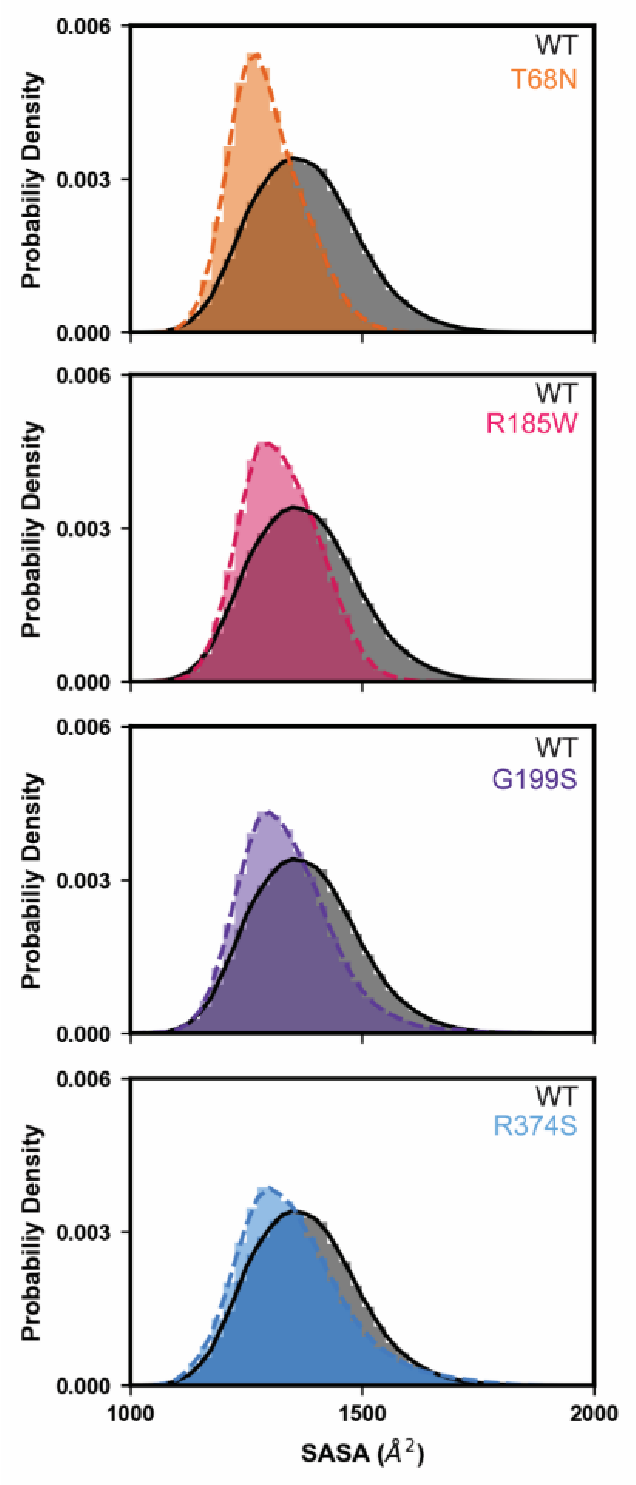

Table S1. *ACTC1* and *ACTA1* missense variants reported as pathogenic in individuals with phenotypes involving cardiac muscle, skeletal muscle, or both. This table contains all missense variants in *ACTA1* or *ACTC1* that were reported as Likely Pathogenic or Pathogenic in ClinVar; Damaging in HGMD, and/or non-synonymous in the *ACTC1* or *ACTA1* LOVD Locus Specific Databases. ACTA1 and ACTC1 are both 377 residues long and differ at only 4 sites (4, 5, 301, 360). Each variant is listed alongside the reported phenotype (one row per affected individual/database entry), whether the affected individual had skeletal muscle or cardiac muscle findings, the Pubmed PMID of any associated publication and/or submitter. The residue numbering listed for ACTA1 variants was updated to current (2022) numbering if necessary.

Table S1 is provided as an Excel spreadsheet.

Table S2. Reported residue-residue pair interaction frequencies that differed among the WT and mutant simulations. This table enumerates the average percent simulation time for which select residue-residue pairs formed inter-residue interactions in the WT and mutant MD simulations. Altered residue-residue interactions are only reported if there was at least a 10% difference in the average contact time frequency between the WT and mutant simulations and a statistically significant difference in the average WT and mutant interaction frequencies.

Table S2 is also provided as an Excel spreadsheet.

Variant	Residue I	Residue J	Contact Frequency (WT, %)	Contact Frequency (Mutant, %)	T-test Statistic	P-Value
T68N	3	103	26	4	2.834	0.047
T68N	14	121	92	80	5.711	0.005
T68N	39	46	7	59	-3.79	0.019
T68N	39	49	17	70	-4.47	0.011
T68N	39	52	42	8	3.805	0.019
T68N	39	53	85	19	7.6	0.002
T68N	40	51	33	0	3.085	0.037
T68N	41	51	13	0	5.527	0.005
T68N	41	65	74	87	-3.475	0.025
T68N	41	67	82	94	-3.185	0.033
T68N	42	51	23	0	3.551	0.024
T68N	43	51	47	3	4.354	0.012
T68N	44	51	27	0	4.167	0.014
T68N	45	49	24	76	-2.977	0.041
T68N	46	51	24	5	5.234	0.006
T68N	47	51	33	5	4.41	0.012
T68N	50	54	16	87	-5.7	0.005
T68N	51	54	13	88	-8.014	0.001
T68N	51	82	8	88	-7.869	0.001
T68N	51	83	6	59	-7.649	0.002
T68N	51	86	6	84	-7.427	0.002
T68N	52	86	22	88	-7.062	0.002
T68N	75	163	85	73	2.923	0.043
T68N	92	131	8	18	-2.846	0.047
R185W	7	355	64	18	4.728	0.009
R185W	8	355	38	2	3.4	0.027
R185W	9	355	57	4	4.689	0.009
R185W	10	92	84	96	-5.449	0.006
R185W	10	358	84	67	7.614	0.002
R185W	11	105	40	26	2.937	0.043
R185W	12	91	54	81	-6.932	0.002

**R185W**	12	92	76	50	5.995	0.004
**R185W**	12	105	56	37	4.637	0.01
**R185W**	14	19	86	72	7.18	0.002
**R185W**	14	76	8	42	-3.143	0.035
**R185W**	16	74	6	79	-5.433	0.006
**R185W**	16	75	12	85	-5.969	0.004
**R185W**	16	77	1	19	-3.258	0.031
**R185W**	16	185	100	0	726.152	0
**R185W**	17	36	25	13	3.079	0.037
**R185W**	17	185	99	0	295.7	0
**R185W**	19	34	72	85	-3.348	0.029
**R185W**	19	84	73	47	3.512	0.025
**R185W**	19	88	43	12	10.62	0
**R185W**	21	92	22	8	7.167	0.002
**R185W**	23	96	74	88	-5.732	0.005
**R185W**	23	103	73	50	3.082	0.037
**R185W**	25	347	51	39	4.542	0.01
**R185W**	31	91	40	21	5.952	0.004
**R185W**	32	58	6	27	-6.528	0.003
**R185W**	32	95	60	36	6.647	0.003
**R185W**	34	71	1	15	-7.819	0.001
**R185W**	34	87	88	68	3.949	0.017
**R185W**	35	73	63	89	-3.77	0.02
**R185W**	35	84	24	9	3.931	0.017
**R185W**	37	86	49	87	-6.088	0.004
**R185W**	37	87	87	97	-3.547	0.024
**R185W**	38	56	67	80	-3.033	0.039
**R185W**	39	46	7	68	-4.233	0.013
**R185W**	39	49	17	67	-3.824	0.019
**R185W**	39	52	42	4	5.082	0.007
**R185W**	39	53	85	38	3.823	0.019
**R185W**	39	70	40	64	-3.301	0.03
**R185W**	40	45	21	90	-4.736	0.009
**R185W**	40	46	23	91	-5.749	0.005
**R185W**	41	46	5	42	-4.787	0.009
**R185W**	41	64	50	66	-7.799	0.001
**R185W**	41	65	74	88	-3.734	0.02
**R185W**	41	67	82	95	-3.446	0.026
**R185W**	42	45	58	100	-5.57	0.005
**R185W**	42	48	14	0	3.239	0.032
**R185W**	43	51	47	9	2.922	0.043
**R185W**	44	51	27	2	3.709	0.021
**R185W**	45	49	24	74	-2.879	0.045
**R185W**	46	51	24	9	3.073	0.037
**R185W**	47	51	33	2	6.377	0.003
**R185W**	48	51	39	0	11.718	0

**R185W**	50	54	16	68	-3.051	0.038
**R185W**	51	54	13	67	-3.625	0.022
**R185W**	51	82	8	48	-4.509	0.011
**R185W**	51	85	1	30	-2.847	0.047
**R185W**	51	86	6	71	-4.372	0.012
**R185W**	52	85	2	18	-5.681	0.005
**R185W**	56	83	35	7	4.063	0.015
**R185W**	56	90	89	99	-2.79	0.049
**R185W**	57	90	13	37	-2.934	0.043
**R185W**	61	71	9	29	-7.417	0.002
**R185W**	70	83	38	63	-7.659	0.002
**R185W**	71	80	44	25	3.283	0.03
**R185W**	71	159	67	24	9.272	0.001
**R185W**	71	185	98	18	4.678	0.009
**R185W**	71	208	54	1	5.683	0.005
**R185W**	73	185	22	0	6.856	0.002
**R185W**	74	185	20	0	8.777	0.001
**R185W**	75	163	85	38	6.651	0.003
**R185W**	75	179	98	75	27.142	0
**R185W**	75	181	78	19	6.857	0.002
**R185W**	76	161	81	12	5.399	0.006
**R185W**	76	163	45	3	9.229	0.001
**R185W**	87	92	22	11	7.734	0.002
**R185W**	88	93	74	62	5.318	0.006
**R185W**	92	105	81	94	-5.211	0.006
**R185W**	93	128	50	33	3.715	0.021
**R185W**	100	104	39	20	5.873	0.004
**R185W**	100	125	19	6	24.044	0
**R185W**	104	355	32	3	3.012	0.039
**R185W**	106	136	86	60	7.426	0.002
**R185W**	106	137	90	72	5.361	0.006
**R185W**	106	348	49	87	-5.507	0.005
**R185W**	106	354	85	51	3.307	0.03
**R185W**	109	377	5	43	-2.974	0.041
**R185W**	110	163	78	19	4.076	0.015
**R185W**	118	376	0	53	-5.678	0.005
**R185W**	118	377	0	55	-5.725	0.005
**R185W**	134	357	18	52	-4.789	0.009
**R185W**	135	348	10	50	-7.664	0.002
**R185W**	135	372	56	3	2.792	0.049
**R185W**	135	374	61	1	3.531	0.024
**R185W**	136	376	1	64	-10.241	0.001
**R185W**	137	348	22	47	-5.38	0.006
**R185W**	137	376	31	1	3.251	0.031
**R185W**	138	377	7	47	-2.838	0.047
**R185W**	140	156	76	61	4.604	0.01

**R185W**	140	341	67	46	2.932	0.043
**R185W**	141	376	18	0	3.437	0.026
**R185W**	141	377	87	23	13.812	0
**R185W**	145	170	11	32	-3.201	0.033
**R185W**	145	377	72	16	5.403	0.006
**R185W**	150	377	27	4	3.163	0.034
**R185W**	158	302	31	6	4.475	0.011
**R185W**	159	186	79	30	3.04	0.038
**R185W**	160	185	96	22	23.03	0
**R185W**	160	186	70	87	-3.516	0.025
**R185W**	163	177	6	36	-3.618	0.022
**R185W**	164	299	8	25	-5.792	0.004
**R185W**	169	377	46	9	4.046	0.016
**R185W**	170	377	88	33	7.927	0.001
**R185W**	172	177	95	70	3.896	0.018
**R185W**	183	263	24	13	5.821	0.004
**R185W**	340	345	53	39	4.306	0.013
**R185W**	344	349	43	29	5.604	0.005
**R185W**	348	376	38	0	3.312	0.03
**R185W**	348	377	87	20	11.32	0
**R185W**	349	354	98	65	2.936	0.043
**R185W**	349	355	91	60	4.729	0.009
**R185W**	352	356	89	68	5.686	0.005
**R185W**	353	357	94	51	4.696	0.009
**R185W**	354	376	57	1	3.234	0.032
**R185W**	354	377	27	10	5.099	0.007
**R185W**	356	375	0	53	-3.947	0.017
**R185W**	357	374	59	3	4.13	0.014
**R185W**	357	376	93	26	15.169	0
**R185W**	358	374	59	4	4.426	0.011
**R185W**	358	375	0	54	-3.857	0.018
**R185W**	359	374	70	8	5.349	0.006
**R185W**	359	375	33	87	-3.402	0.027
**R185W**	359	376	1	53	-6.749	0.003
**R185W**	360	374	16	2	3.156	0.034
**R185W**	360	375	0	16	-3.055	0.038
**R185W**	363	374	79	30	5.195	0.007
**R185W**	363	375	0	48	-10.399	0
**R185W**	367	374	18	52	-3.945	0.017
**R185W**	370	374	42	95	-4.137	0.014
**R185W**	371	375	35	91	-3.582	0.023
**R185W**	372	376	0	67	-15.053	0
**R185W**	373	376	0	67	-12.103	0
**R185W**	373	377	0	26	-4.84	0.008
**G199S**	25	347	51	40	3.158	0.034
**G199S**	41	51	13	1	4.313	0.013

**G199S**	44	48	25	72	-4.363	0.012
**G199S**	45	48	21	74	-3.325	0.029
**G199S**	45	49	24	80	-3.674	0.021
**G199S**	46	51	24	6	6.68	0.003
**G199S**	104	131	38	28	3.412	0.027
**G199S**	200	248	17	41	-4.916	0.008
**G199S**	259	263	69	81	-3.538	0.024
**R374S**	3	6	20	65	-3.318	0.029
**R374S**	3	30	39	7	3.421	0.027
**R374S**	4	355	2	15	-4.836	0.008
**R374S**	7	349	32	18	3.239	0.032
**R374S**	7	355	64	41	2.83	0.047
**R374S**	8	24	30	65	-5.097	0.007
**R374S**	8	104	89	78	3.383	0.028
**R374S**	39	53	85	50	2.845	0.047
**R374S**	41	65	74	87	-3.406	0.027
**R374S**	43	51	47	7	3.417	0.027
**R374S**	44	51	27	2	3.829	0.019
**R374S**	45	53	0	14	-4.01	0.016
**R374S**	48	51	39	9	4.221	0.013
**R374S**	51	54	13	58	-5.033	0.007
**R374S**	51	82	8	51	-3.546	0.024
**R374S**	51	85	1	29	-9.405	0.001
**R374S**	51	86	6	51	-5.115	0.007
**R374S**	52	86	22	63	-2.852	0.046
**R374S**	56	83	35	13	2.895	0.044
**R374S**	100	104	39	24	3.798	0.019
**R374S**	100	125	19	7	4.532	0.011
**R374S**	104	131	38	23	3.052	0.038
**R374S**	106	348	49	75	-3.559	0.024
**R374S**	122	136	52	22	3.484	0.025
**R374S**	130	361	66	79	-4.319	0.012
**R374S**	135	372	56	0	2.985	0.041
**R374S**	135	374	61	0	3.567	0.023
**R374S**	135	377	44	93	-2.788	0.049
**R374S**	137	348	22	39	-4.015	0.016
**R374S**	137	376	31	0	3.302	0.03
**R374S**	141	376	18	0	3.437	0.026
**R374S**	145	170	11	23	-3.157	0.034
**R374S**	145	377	72	36	2.936	0.043
**R374S**	150	377	27	3	3.343	0.029
**R374S**	169	377	46	15	3.478	0.025
**R374S**	348	376	38	0	3.312	0.03
**R374S**	354	376	57	7	2.86	0.046
**R374S**	357	374	59	0	4.338	0.012
**R374S**	358	363	65	24	3.391	0.028

**R374S**	358	374	59	0	4.751	0.009
**R374S**	359	363	76	44	3.305	0.03
**R374S**	359	364	64	28	2.914	0.043
**R374S**	359	375	33	91	-3.972	0.017
**R374S**	360	374	16	0	3.604	0.023
**R374S**	363	374	79	2	11.273	0
**R374S**	370	374	42	91	-3.838	0.018
**R374S**	371	375	35	96	-4.043	0.016
**R374S**	372	375	43	100	-2.856	0.046
