# Supplementary material for: Variants in ACTC1 underlie distal arthrogryposis accompanied by congenital heart defects
Source: HGG Adv. 2023 Jun 15;4(3):100213. doi: 10.1016/j.xhgg.2023.100213 (PMC10345160; doi:10.1016/j.xhgg.2023.100213)
Supplement: Document S2. Article plus supplemental information [file mmc4.pdf]

HGG
Advances

ARTICLE

Variants in *ACTC1* underlie distal arthrogryposis accompanied by congenital heart defects

Jessica X. Chong,[1,2,15] Matthew Carter Childers,[3,4,15] Colby T. Marvin,[1] Allison J. Marcello,[1] Hernan Gonorazky,[5] Lili-Naz Hazrati,[6] James J. Dowling,[5,7] Fatema Al Amrani,[5,8] Yasemin Alanay,[9] Yolanda Nieto,[10] Miguel Á Marín Gabriel,[11] Arthur S. Aylsworth,[12] Kati J. Buckingham,[1] Kathryn M. Shively,[1] Olivia Sommers,[1] Kailyn Anderson,[1] University of Washington Center for Mendelian Genomics, University of Washington Center for Rare Disease Research, Michael Regnier,[3,4] and Michael J. Bamshad[1,2,4,13,14,16,*]

Summary

Contraction of the human sarcomere is the result of interactions between myosin cross-bridges and actin filaments. Pathogenic variants in genes such as *MYH7*, *TPM1*, and *TNNI3* that encode parts of the cardiac sarcomere cause muscle diseases that affect the heart, such as dilated cardiomyopathy and hypertrophic cardiomyopathy. In contrast, pathogenic variants in homologous genes such as *MYH2*, *TPM2*, and *TNNI2* that encode parts of the skeletal muscle sarcomere cause muscle diseases affecting skeletal muscle, such as distal arthrogryposis (DA) syndromes and skeletal myopathies. To date, there have been few reports of genes (e.g., *MYH7*) encoding sarcomeric proteins in which the same pathogenic variant affects skeletal and cardiac muscle. Moreover, none of the known genes underlying DA have been found to contain pathogenic variants that also cause cardiac abnormalities. We report five families with DA because of heterozygous missense variants in the gene *actin, alpha, cardiac muscle 1* (*ACTC1*). *ACTC1* encodes a highly conserved actin that binds to myosin in cardiac and skeletal muscle. Pathogenic variants in *ACTC1* have been found previously to underlie atrial septal defect, dilated cardiomyopathy, hypertrophic cardiomyopathy, and left ventricular noncompaction. Our discovery delineates a new DA condition because of variants in *ACTC1* and suggests that some functions of *ACTC1* are shared in cardiac and skeletal muscle.

Introduction

Sarcomeres are the repeating functional units of muscle cells that are joined end to end to form skeletal and cardiac muscle fibers.[1] Sarcomeres consist of thick myosin filaments and thin actin filaments along with proteins such as troponin and tropomyosin that facilitate and regulate the interactions between the filaments.[2] Contractile force is generated when the myosin and actin filaments bind to form cross-bridges, which is thought to cause the filaments to slide against each other. The key sarcomeric proteins are encoded by highly conserved and homologous genes that typically express an isoform that is predominant in either cardiac or skeletal muscle. For example, *TNNI2*, *MYH3*, and *ACTA1* encode isoforms of troponin, myosin heavy chain, and α-actin, respectively, that are primarily expressed in skeletal muscle, while *TNNI3*, *MYH6*, and *ACTC1* encode isoforms that are primarily expressed in cardiac muscle.[3]

Pathogenic variants in genes that encode the skeletal sarcomeric proteins tropomyosin (*TPM2*; MIM: 190990);[4] troponin I2, fast skeletal type (*TNNI2*; MIM: 191043);[4] troponin T3, fast skeletal type (*TNNT3*; MIM: 600692);[5] myosin heavy chain 3 (*MYH3*; MIM:160720);[6] and myosin heavy chain 8 (*MYH8*; MIM: 160741)[7] account for most cases of distal arthrogryposis (DA), a group of Mendelian conditions characterized by non-progressive congenital contractures of the limbs and, less frequently, contractures of the face, strabismus, neck webbing, pterygia, short stature, and scoliosis. The precise pathogenesis of the contractures is unknown, although it has been proposed that pathogenic variants lead to perturbation of muscle contraction or relaxation, resulting in reduced limb movement *in utero*.[8,9] Several additional genes, specifically *PIEZO2* [MIM: 613629],[10,11] *ECEL1* [MIM: 605896],[12,13] and *FBN2* [MIM: 612570],[14] that underlie other forms of DA encode proteins that are less directly involved in sarcomere contraction.

[1]Division of Genetic Medicine, Department of Pediatrics, University of Washington, Seattle, WA 98195, USA; [2]Brotman-Baty Institute, Seattle, WA 98195, USA; [3]Department of Bioengineering, University of Washington, Seattle, WA 98195, USA; [4]University of Washington Center for Translational Muscle Research, Seattle, WA 98195, USA; [5]Division of Neurology, The Hospital for Sick Children, Toronto, ON M5G 1X8, Canada; [6]Department of Paediatric Laboratory Medicine, The Hospital for Sick Children, Toronto, ON M5G 1X8, Canada; [7]Departments of Paediatrics and Molecular Genetics, University of Toronto, Toronto, ON M5G 0A4, Canada; [8]Division of Neurology, Department of Pediatrics, Sultan Qaboos University Hospital, Sultan Qaboos University, Muscat, Sultanate of Oman; [9]Division of Pediatric Genetics, Department of Pediatrics, School of Medicine, Acibadem Mehmet Ali Aydinlar University, 34752 Istanbul, Turkey; [10]Department of Basic Bio-Medical Sciences, European University of Madrid, Madrid, Spain; [11]Department of Pediatrics, Puerta de Hierro-Majadahonda University Hospital, 28221 Madrid, Spain; [12]Departments of Pediatrics and Genetics, University of North Carolina, Chapel Hill, NC 27599, USA; [13]Department of Genome Sciences, University of Washington, Seattle, WA 98195, USA; [14]Seattle Children's Hospital, Seattle, WA 98105, USA
[15]These authors contributed equally
[16]Lead contact
*Correspondence: mbamshad@uw.edu
https://doi.org/10.1016/j.xhgg.2023.100213.
© 2023 The Author(s). This is an open access article under the CC BY license (http://creativecommons.org/licenses/by/4.0/).

To date, the vast majority of variants in genes encoding homologous components of the cardiac sarcomere have been found to result in conditions in which only cardiac muscle is affected, including cardiomyopathy and structural heart defects. Here, we report five families with DA and congenital heart defects because of heterozygous missense variants in the gene *actin*, *alpha*, *cardiac muscle 1* (*ACTC1*). *ACTC1* encodes a highly conserved actin that binds to myosin in cardiac and skeletal muscle. We employ molecular dynamics (MD) simulations of wild-type (WT) and mutant cardiac actin to predict the structural and functional consequences of these variants.

Methods

Exome sequencing, annotation, and filtering

From a cohort of 463 families (1,582 individuals) with multiple congenital contractures, we selected 172 families in which pathogenic or likely pathogenic variants had not been identified, for exome sequencing (ES). All studies were approved by the institutional review boards of the University of Washington and Seattle Children's Hospital, and informed consent was obtained from each participant or their parents. ES, annotation, and analysis were performed by the University of Washington Center for Mendelian Genomics (now the University of Washington Center for Rare Disease Research) as described previously.[15] Briefly, variants were called by GATK v.3.7 HaplotypeCaller and annotated with Variant Effect Predictor v.95.3.[16] Variants were filtered using GEMINI v.0.30.1[17] for genotype call quality (GQ $\geq$ 20), read depth ($\geq$6), allele frequency in population controls (i.e., maximum frequency in any continental superpopulation in gnomAD[18] v.2.1 and v.3.0 exomes and genomes $<$ 0.005), consistency with the mode of inheritance in each family, and predicted impact on protein-coding sequence (e.g., annotated as missense, nonsense, canonical splice, or coding insertion or deletion [indel]).

MD: Model preparation and simulation

Initial coordinates for cardiac globular (g-actin) structures were generated via homology to an X-ray crystal structure of rabbit skeletal actin (UniProt: P68135) downloaded from the Protein Data Bank[19] (www.rcsb.org; PDB: 3HBT).[20] PDB: 3HBT is a model of g-actin complexed with ATP, Ca^{2+}, and SO_4. The human ACTC1 sequence was downloaded from UniProt (P68032). The human and rabbit sequences were 98.9% identical, as assessed using Clustal Omega,[21] and there were four conservative amino acid substitutions (human amino acid, human residue number, and rabbit amino acid as follows: Asp2Glu, Glu3Asp, Leu301Met, and Ser360Thr). Homology models of the human WT and four mutant (p.Thr68Asn, p.Arg185Trp, p.Gly199Ser, and p.Arg374Ser) structures were generated using Modeller,[22] which introduced the amino acid substitutions and built coordinates for atoms not present in the PDB file (no coordinates were present for D-loop residues 40–50 in PDB: 3HBT). The p.Arg374His variant was not simulated because of the expected overlap with p.Arg374Ser and because the change to His is more conservative than the change to Ser. For p.Gly199Ser, the initial backbone dihedral (φ, ψ) angles for Gly were (152°, −16°), which are unfavorable for Ser. Consequently, this loop was further refined, and the initial S199 dihedrals were (−177°, −14°). During modeling, crystallographic waters and the SO_4 were removed, ATP was retained, and Ca^{2+} was replaced by Mg^{2+}. Initial coordinates for cardiac filamentous (F-actin) pentamer structures were generated using an electron microscopy structure of mouse tropomyosin and rabbit skeletal actin (PDB: 3J8A).[23] The tropomyosin chains were removed, and the F-actin pentamers complexed with ADP and Mg^{2+} were used to construct homology models of WT and p.Thr68Asn human cardiac F-actin with Modeller. After homology models were built, hydrogen atoms were modeled onto the initial structure using the tleap module of AMBER, and each protein was solvated with explicit water molecules in a periodic, truncated octahedral box that extended 10 Å beyond any protein atom. Na^+ counterions were added to neutralize the systems.

All simulations were performed with the AMBER20 package[24,25] and the ff14SB force field[26] using standard procedures. Water molecules were treated with the TIP3P force field.[27] Metal ions were modeled using the Li and Merz parameter set.[28–30] ATP and ADP molecules were treated with parameters from Meagher et al.[31] The SHAKE algorithm was used to constrain the motion of hydrogen-containing bonds. Long-range electrostatic interactions were calculated using the particle mesh Ewald (PME) method. Each system was minimized in 10,000 steps divided across 3 stages in which restraints were placed either on hydrogen atoms, solvent atoms, or all backbone heavy atoms (C_α, C, N, O atoms). After minimization, systems were heated to 310 K over 300 ps using the canonical NVT (constant number of particles, volume, and temperature) ensemble. During all heating stages, 25 kcal mol^{-1} restraints were present on the backbone heavy atoms (C_α, C, N, O atoms). After the system temperature reached 310 K, the systems were equilibrated for 5.4 ns over 5 successive stages using the NPT (constant number of particles, pressure, and temperature) ensemble. During equilibration, restraints on backbone atoms were decreased from 25 kcal mol^{-1} during the first stage to 1 kcal mol^{-1} during the fourth stage. During the final equilibration stage, the systems were equilibrated in the absence of restraints. Production dynamics for conventional MD (cMD) simulations were then performed using the NVT ensemble using an 8-Å nonbonded cutoff and a 2-fs time step, and coordinates were saved every picosecond. G-actin cMD simulations were run in triplicate; each replicate simulation was 500 ns long. We used an enhanced sampling scheme called Gaussian accelerated MD (GaMD)[32] to explore conformational sampling in the WT and p.Thr68Asn F-actin pentamer models. GaMD production runs were preceded by a 52-ns-long GaMD equilibration period in which boost potentials were added. The upper limits of the SD of the boost potentials were set to 6 kcal mol^{-1}. Neither the standard 5.2-ns equilibration nor the 52-ns GaMD equilibration contributed to the length of the production dynamics for any simulation. Production dynamics for GaMD simulations were using the NVT ensemble using an 8-Å nonbonded cutoff and a 2-fs time step, and coordinates were saved every picosecond. Single replicates of the WT and p.Thr68Asn were performed, and each simulation was 300 ns long. Unless specified otherwise, simulations were analyzed separately, and the results of replicate simulations were averaged together.

MD: Analysis

The C_α root-mean-square deviation (RMSD), C_α root-mean-squared fluctuation (RMSF), solvent-accessible surface area (SASA), secondary structure content, inter-atomic distances, and inter-residue contacts were calculated with cpptraj.[33] The C_α RMSD was calculated after alignment of all C_α atoms to the

Table 1. Clinical findings of individuals with DA because of heterozygous variants in *ACTC1*

Family		A	A	B	B	C	C	D	E
Ancestry		Turkish	Turkish	European	European	European	European	European	European
Individual		II-1	III-1	II-2	III-1	II-2	III-1	II-1	II-1
Sex		male	female	female	female	female	male	male	male
Age at last assessment (years)		59	18	53	24	27	5	16	stillbirth (38+ 3 weeks)
**Variant**									
ClinGen allele ID		CA391630889	CA391630889	CA391628517	CA391628517	CA391631962	CA391631962	CA391631962	CA391628507
cDNA change (NM_005159.4)		c.595G>A	c.595G>A	c.1120C>A	c.1120C>A	c.203C>A	c.203C>A	c.553C>T	c.1121G>A
Genomic coordinates (hg19)		chr15:g. 35084630C>T	chr15:g. 35084630C>T	chr15:g. 35082627G>T	chr15:g. 35082627G>T	chr15:g. 35085697 G>T	chr15:g. 35085697 G>T	chr15:g. 35084672G>A	chr15:g. 35082626C>T
Predicted amino acid change		p.Gly199Ser	p.Gly199Ser	p.Arg374Ser	p.Arg374Ser	p.Thr68Asn	p.Thr68Asn	p.Arg185Trp	p.Arg374His
Zygosity		het	het	het	het	het	het	het	het
Inheritance		unknown	inherited	inherited	inherited	de novo	inherited	de novo	de novo
CADD score (v.1.6)		25.6	25.6	23.7	23.7	24.7	24.7	27.9	25.5
Allele count (gnomAD v.2.1.1 + v.3.1.2)		0	0	0	0	0	0	1	0
Original diagnosis		autosomal dominant multiple pterygium syndrome	autosomal dominant multiple pterygium syndrome	Sheldon-Hall syndrome	Sheldon-Hall syndrome	Freeman-Sheldon syndrome	N/D	multiple pterygium syndrome and cardiomyopathy	–
**Clinical features: Growth**									
Weight percentile		57 (72 kg)	<1 (40 kg) −2.95 SDS	78 (70 kg)	<1 (37 kg)	2 (121 kg)	1 (13.6 kg)	<3 (29 kg)	33 (3050 g)
Height percentile		<1 (155 cm)	<1 (134 cm) −4.86 SDS	<1 (147 cm)	<1 (140 cm)	56 (163 cm)	4 (102cm)	<3 (149 cm)	N/A
Short stature	HP:0004322	+	+	+	+	–	+	+	N/A
**Clinical features: Limbs**									
Knee contractures	HP:0006380	–	+	+	+	–	N/D	+	+
Vertical talus	HP:0001838	–	+	–	–		N/D	N/D	+
Equinovarus/clubfoot	HP:0001762	–	+	+ (bilateral)	+ (bilateral)	+	N/D	+	+

(*Continued on next page*)

Table 1. *Continued*

Family		A	A	B	B	C	C	D	E
Camptodactyly, toes	HP:0001836	–	–	+	+	+	N/D	N/D	+
Elbow contractures	HP:0002987	+	–	N/D	–	–	+	+	+
Webbed elbow	HP:0009760	–	–	N/D	N/D	–	+	+	N/D
Limited forearm supination	HP:0006394	+	+	+	+	–	N/D	+	+
Contractures of wrists	HP:0001239	–	–	+	+	–	N/D	–	+
Camptodactyly, fingers	HP:0100490	+	+	+	+	+	+	N/D	+
Hypoplastic flexion creases		+	+	+	+	+	N/D	+	+
**Clinical features: Face and neck**									
Downslanting palpebral fissures	HP:0000494	+	+	–	–	+	+	+	+
Ptosis	HP:0000508	+	+	+	+	+	–	+	+
Downturned corners of the mouth	HP:0002714	+	+	+	+	+	+	N/D	N/D
Microretrognathia	HP:0000308	+	+	+	+	+	N/D	+	+
Long nasal bridge		+	+	+	+	+	+	N/D	+
Low-set ears	HP:0000369	+	+	+	+	+	+	N/D	+
Limited neck rotation	HP:0005986	+ no vertebral fusion	+ no vertebral fusion	+	+	+	N/D	+ (congenital torticollis)	+
Webbed neck	HP:0000465	+ mild	+ profound	+	+	+	+	N/D	+
**Other**									
Scoliosis	HP:0002650	+ mild	+	+ restrictive lung disease (HP:0002091)	+	N/D	–	+	–
Hip contractures	HP:0003273	+	+	+	+	N/D	–	+	+
Cardiomyopathy	HP:0001638	–	–	N/D	N/D	N/D	N/D	+ (dilatated)	– (fetal US)
Atrial septal defect	HP:0001631	+	+	N/D	N/D	+	+	+	– (fetal US)
Ventricular septal defect	HP:0001629	–	–	N/D	N/D	N/D	–	+	– (fetal US)

(*Continued on next page*)

Table 1. *Continued*

Family	A	A	B	B	C	C	D	E
Other features	high myopia, glaucoma, retinal detachment	mitral valve repair, myopia	bilateral fixed knee extension/limited knee extension (HP:0003066)	iris coloboma (HP:0000612) of the left eye, unilateral conductive hearing impairment (HP:0040119), stapedectomy at age 8		non-verbal (HP: 0001344), autism spectrum disorder (HP:0000729), widely spaced nipples (HP: 0006610), anteverted ears (HP:0040080), flat foot (HP:0001763)	cryptorchidism, arachnoid cyst on base of skull, cleft palate	ductus venosus agenesis (HP:003419), hydrops fetalis (HP:000178), hydrothorax (HP: 002567), mild polyhydramnios (HP:0001561)

Plus (+) indicates presence of a finding, and minus (−) indicates absence of a finding. *, described per report; N/D, no data were available; N/A, not applicable; CADD, Combined Annotation Dependent Depletion v.1.6. cDNA positions are named using HGVS notation and GenBank: NM_005159.4. Predicted amino acid changes are shown. US, ultrasound.

minimized structure. The C_α RMSF was calculated about average MD structures for each simulation. For each time point in the simulation, two residues were considered to be in contact with one another when at least one pair of heavy atoms was within 5 Å of another. Then we recorded the average percent simulation time each residue pair was in contact for each simulation. A Student's t test was used to identify statistically significant ($p < 0.05$) differences in inter-residue contact times between the WT and mutant simulations. All protein images were prepared using UCSF Chimera.[34,35]

Results

After variant filtration of the exome data, three families had compelling candidate variants in the same candidate gene, *ACTC1* (MIM 102540; GenBank: NM_005159.4) (Table 1; Figures 1 and S1). Specifically, each family had a heterozygous candidate missense variant that was either *de novo* or segregated in an autosomal dominant pattern. In family A, comprised of an affected father and affected daughter with camptodactyly of the fingers, hypoplastic flexion creases, clubfoot, webbed neck, scoliosis, hip contractures, and ventriculoseptal defect, a heterozygous variant in *ACTC1* (c.595G>A, p.Gly199Ser) was identified. This family has been described previously (family D in Chong et al.[36]) as possibly having autosomal dominant multiple pterygium syndrome (MIM: 178110), but no likely pathogenic or pathogenic variants in *MYH3* were identified. In family B, an affected mother and affected daughter with knee contractures, clubfoot, limited neck rotation, scoliosis, and hip contractures were heterozygous for c.1120C>A, p.Arg374Ser. The grandmother in family B was described as having similar clinical findings, but no medical records or photographs were available. Family C, the third family, was a simplex family in which a *de novo* variant, c.203C>A, p.Thr68Asn, was identified in the proband with clubfoot, camptodactyly of the toes and fingers, webbed neck, and an atrial septal defect. Upon follow-up with family C approximately 20 years after they were originally enrolled, the proband was found to have a son who had camptodactyly of the fingers, overlapping toes, elbow contractures, elbow webbing, and an atrial septal defect. Her son was heterozygous for the c.203C>A, p.Thr68Asn variant. No other variants of interest were identified in any of these families in genes known to underlie DA or congenital heart defects.

Two additional simplex families (families D and E) subsequently came to our attention via direct referral after clinical testing identified *ACTC1* as a candidate gene. Each had a *de novo* variant: c.553C>T, p.Arg185Trp in family D and c.1121G>A, p.Arg374His in family E. Notably, the variants in families B and E perturbed the same residue. The proband of family D had short stature, clubfoot, knee and elbow contractures, hypoplastic flexion creases, atrial septal defect, and ventricular septal defect. No muscle weakness was noted. An echocardiogram found borderline left ventricular systolic function with an ejection fraction

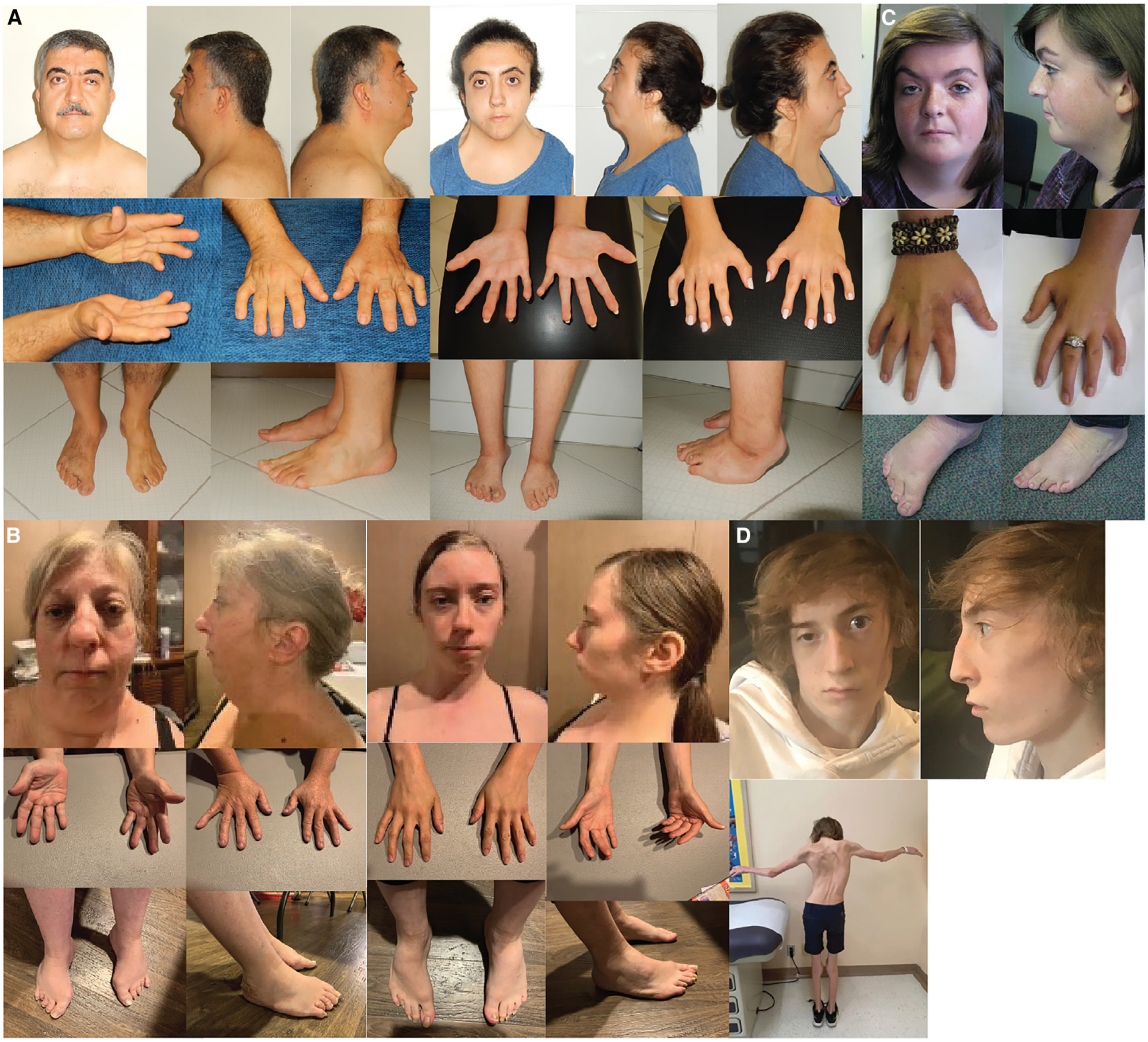

Figure 1. Phenotypic characteristics of individuals with DA because of heterozygous variants in *ACTC1*
(A–D) The characteristics shown include webbed neck, bilateral clubfoot, camptodactyly of the fingers, and hypoplastic flexion creases in family A (A; II-1 and III-1); camptodactyly, webbed neck, bilateral clubfoot, camptodactyly of the fingers and toes, and hypoplastic flexion creases in family B (B; II-2 and III-1); webbed neck, bilateral clubfoot, webbed neck, bilateral clubfoot, and camptodactyly of the fingers and toes in family C (C; II-2); and ptosis, webbed neck, camptodactyly of the fingers, and scoliosis in family D (D; II-1). Table 1 contains a detailed description of the clinical findings of each affected individual, and Figure S1 provides a pedigree for each family.

of 52% and reduced longitudinal strain in the basal wall segments. Electron microscopy and immunohistochemistry of a skeletal muscle biopsy revealed possible nemaline bodies (Figure S2) and the presence of rare myofibers with a central core but no ragged red fibers or rod-like inclusions. The proband in family E was stillborn at 38+3 weeks. At 31 weeks, ultrasound findings included hydrops fetalis, hydrothorax, a small lower jaw, ductus venosus agenesis, and positioning of the extremities consistent with fetal akinesia. No additional clinical information was available.

In summary, at least one affected individual in each family was reported to have a combination of camptodactyly of the fingers or toes, hypoplastic flexion creases, clubfoot, limited neck rotation, scoliosis (excluding family E, for which only fetal ultrasound was available), and hip contractures. Common facial features included microretrognathia, ptosis, downslanting palpebral fissures, low-set ears, and a long nasal bridge (Figure 1). Ventricular or atrial septal defects were reported in families A, C, and D, while family E had ductus venosus agenesis *in utero*, but only one affected individual, the proband in D, had cardiomyopathy. The co-occurrence of these congenital heart defects is notable because *ACTC1* is well established to underlie isolated cardiac abnormalities, including dilated and hypertrophic cardiomyopathy (MIM: 613424, 612098), atrial septal defects (MIM: 612794), and left ventricular

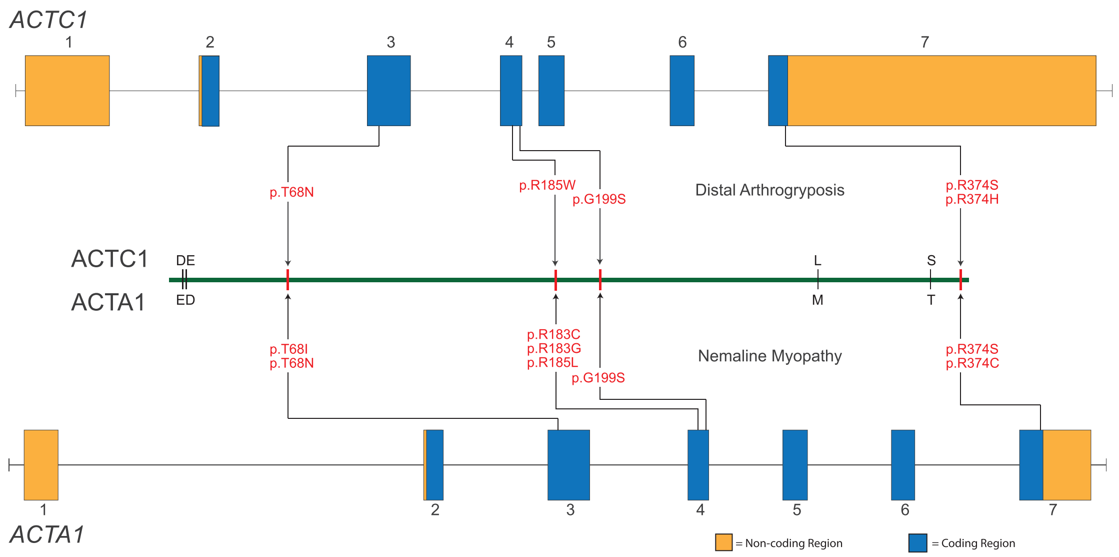

Figure 2. Genomic model of *ACTC1* and *ACTA1*
Illustrated are each of the variants found in ACTC1 that underlie DA and the homologous sites in ACTA1 that result in severe nemaline myopathy when mutated. *ACTC1* and *ACTA1* are each composed of 7 exons and consist of protein-coding (blue) and non-coding (orange) sequences. The proteins are nearly identical except for four residues (represented by single-letter amino acid codes immediately above and below the green line). The approximate location of each pathogenic variant (red text) is indicated by an arrow.

noncompaction (MIM: 613424). However, *ACTC1* has not been reported to underlie a multiple malformation syndrome that affects multiple organs.

Combined Annotation Dependent Depletion (CADD; v.1.6)[37] scores greater than 20.0 indicate that all five variants are predicted to be pathogenic (Table 1). For all five of these variants, the homologous residues in ACTA1 have been reported[38–43] to be perturbed in infants with autosomal dominant severe congenital nemaline myopathy (Figure 2), leading to death before 1 year of age, an observation that further suggests that these residues play a critical role in sarcomere function. In addition, these variants were either absent or exceedingly rare in gnomAD v.2.1.1 or v.3.1.2. p.Arg185Trp was heterozygous in a single individual in gnomAD and was the only variant that has been reported previously in ClinVar (twice classified as "likely pathogenic"). One of these ClinVar entries (SCV000742090.2) reports that p.Arg185Trp was found in an individual with "arthrogryposis multiplex congenita, multiple suture craniosynostosis, high palate, cleft uvula, pulmonary hypoplasia, bronchomalacia, pulmonary arterial hypertension, hydrocephalus, cryptorchidism, penile hypospadias, dysphagia, secundum atrial septal defect, patent foramen ovale, shallow orbits, infra-orbital crease, microretrognathia, webbed neck, short neck." These clinical findings suggest that this individual likely has the same condition we describe here.

Pathogenic variant-associated changes in the overall conformation and dynamics of g-actin

We first analyzed the conformations sampled by WT and mutant g-actin to determine whether four of the variants we identified (Figure 3A) led to large-scale conformational changes within actin monomers. We calculated the C_α RMSD (a measure of structural similarity) of each frame in the simulation to the minimized structure. The overall conformation of the actin monomers was preserved despite introduction of each variant. In fact, the mutant simulations all had smaller C_α RMSD values than the WT simulations, indicating that the variants dampened structural fluctuations in g-actin. All simulated systems had an average C_α RMSD (a measure of structural similarity) of less than 2.6 Å to the crystallographic conformation (Table 2; WT: 2.6 Å, p.p.Thr68Asn: 2.0 Å test statistic = 5.09, p = 0.007, p.Gly199Ser, p.Arg185Trp: 2.4 Å test statistic = 2.00, p = 0.12, p.Gly199Ser: 2.4 Å test statistic = 1.14, p = 0.32, p.Arg374Ser: 2.2 Å test statistic = 5.33, p = 0.006; p values denote statistical differences in the average RMSD values of the WT simulations versus each mutant). The largest-amplitude structural change was a breathing motion in which relative scissoring of SD2 (subdomain 2) and SD4 opened and closed the nucleotide binding pocket, which occurred in all simulations. We next measured the C_α RMSFs and compared them with the WT simulations (Figure S3). The majority of residues in g-actin had small (<1 Å) C_α fluctuations about their average positions. The regions with the greatest fluctuations were the DNase1 binding loop (also known as the D-loop, residues 41–56) and two loops in SD4 (residues 199–204 and 219–224). The flexibilities of most residues were not affected by the variants. However, all four variants led to a decrease in the C_α RMSF of residues in the D-loop (Figure S3), and statistically significant (residues with significant differences denoted in Figure S3) decreases in the C_α RMSF of D-loop residues were observed for p.Thr68Asn, p.Arg185Trp, and p.Gly199Ser. Each of the variants also caused low-magnitude (<0.5 Å)

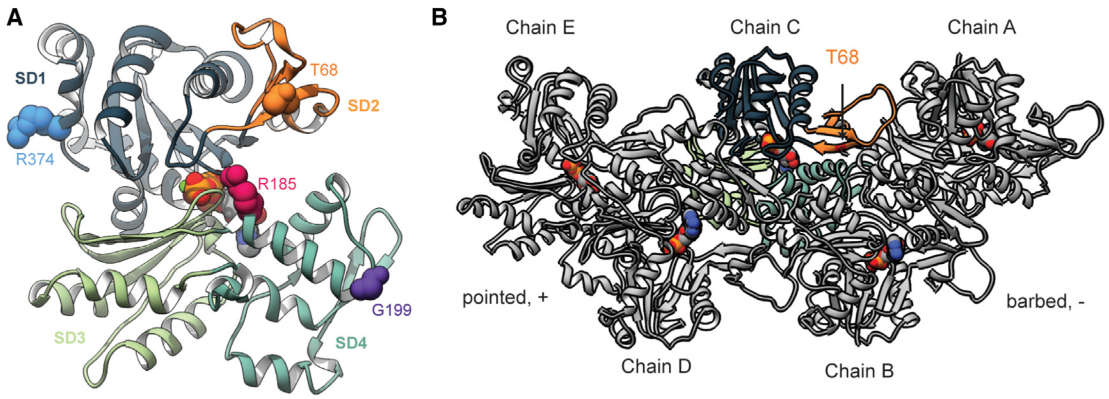

Figure 3. Molecular structures of the globular and filamentous forms of human cardiac actin
(A) Globular actin (g-actin) monomers are comprised of four subdomains (subdomains 1–4 [SD1–SD4]) arranged around the nucleotide binding pocket. The g-actin monomer simulated in this study contains ATP in the binding pocket. The atoms of four residues corresponding to variant sites examined in this study are shown as spheres: T68 (orange), R185 (magenta), G199 (purple), and R374 (blue).
(B) Actin monomers polymerize into protofibrils, which then associate with one another to form filamentous actin (F-actin). In this F-actin pentamer, chains A, C, and E form one protofibril, and chains B and D form the other. The pentamer simulated here has ADP molecules in the nucleotide binding pockets. The location of residue T68 is denoted on chain C.

but statistically significant (residues with significant differences denoted in Figure S3) (p value and test) changes in C_α RMSF among residues near the variant sites.

p.Thr68Asn modified the structural organization of SD2 and the D-loop

The RMSD and RMSF data indicated substantial changes in the dynamics of the D-loop in the presence of all four simulated variants. Therefore, we examined the dynamics in this region in greater detail with an emphasis on the p.Thr68Asn variant because T68 is located within SD2 and closest structurally to the D-loop. Changing Thr to Asn (p.Thr68Asn) is somewhat conservative; both residues have polar side chains, but the Asn side chain is long, whereas Thr branches at C_b. The alternate conformations accessible to Asn led to a cascade of changes in amino acid interactions among neighboring residues in SD2 (Figures 4A–4C). The changes in contacts affected interactions made by D-loop residues as well as a complex salt bridge formed between residues 39, 70, and 83. p.Thr68Asn increased the extent to which residues in the D-loop formed an α helix secondary structure (Figures 4B and 4D). p.Thr68Asn decreased the overall SASA of residues in the D-loop relative to the WT simulations (Figure 4E). The net effect of the structural changes induced by p.Thr68Asn shifted the structure and dynamic behavior of SD2 so that the mutant SD2 adopted a more compact and less flexible conformation relative to the WT.

These effects were most pronounced for p.Thr68Asn but were also observed for the other variants. The greater effect of p.Thr68Asn was likely due to its central position in SD2. Altered structure and dynamics among D loop residues were also observed for the p.Gly199Ser, p.Arg185Trp, and p.Arg374Ser simulations (Figures S4–S6). All variants altered inter-residue interactions formed by D loop residues and other SD2 residues (Figure S4). All variants increased the extent to which residues in the D-loop formed an α helix secondary structure in the ensemble average (p.Arg374Ser>p.Arg185Trp>p.Gly199Ser>p.Thr68Asn>-WT; Figure S5). However, there was not a statistically meaningful change in the net amount of α helix formed, and this region did form an enduring α helix in one of the WT simulations. All variants decreased the average D-loop SASA (WT [1,375 $Å^2$] > p.Arg374Ser [1,349 $Å^2$, t statistic = 0.70, p = 0.52] > p.Gly199Ser [1,335 $Å^2$, t statistic = 1.47, p = 0.21] > p.Arg185Trp [1,325 $Å^2$, t statistic = 3.30, p = 0.03] > p.Thr68Asn [1,294 $Å^2$, t statistic = 3.53, p = 0.02]; Figure S6). We analyzed statistically significant (Figures 4 and S4; Table S2) changes in residue-residue contact networks to identify structural pathways by which the variants altered SD2 dynamics (Figure 5; Table S2). Altered residue-residue interactions were only considered in this analysis when there was at least a 10% difference in the average contact time frequency between the WT and mutant simulations. The extent to which the variants altered residue-residue interaction networks was variable. Disruption was greatest for the p.Arg185Trp variant, and the p.Gly199Ser variant was the least impactful. P.Thr68Asn modified local residue-residue interactions to affect changes in SD2 and the D-loop. p.Arg185Trp, p.Gly199Ser, and p.Arg374Ser instead introduced structural changes that propagated

Table 2. C_α RMSD values for g-actin monomer simulations

System	Run 1	Run 2	Run 3	Average
WT	2.7	2.5	2.5	2.6
p.Thr68Asn	2.1	2.2	1.8	2.0
p.Arg185Trp	2.4	2.5	2.3	2.4
p.Gly199Ser	2.7	2.5	2.1	2.4
p.Arg374Ser	2.4	2.2	2.1	2.2

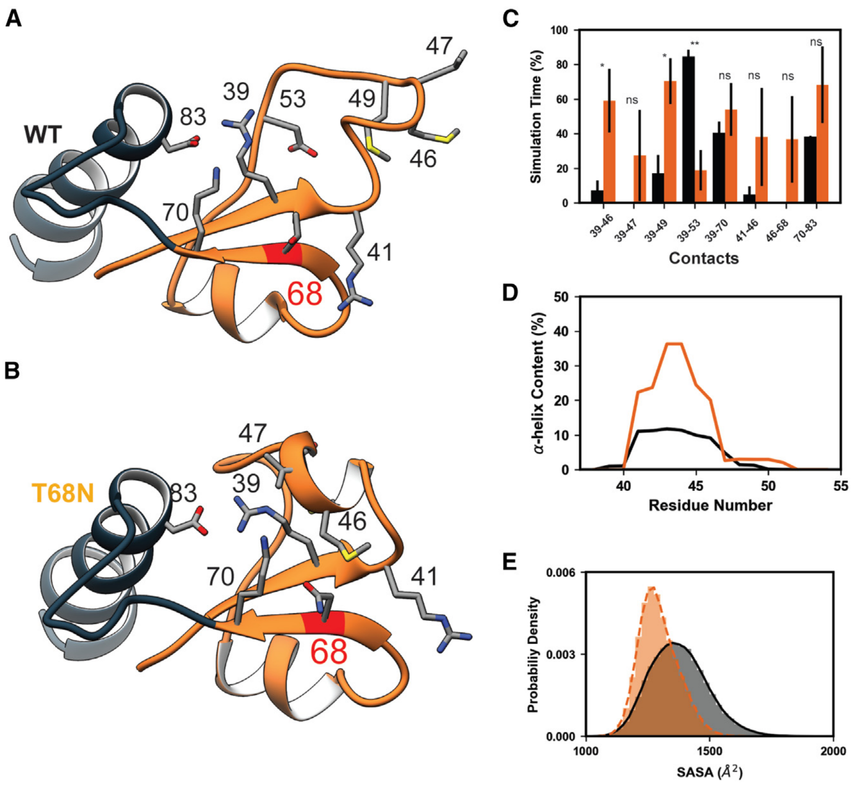

Figure 4. p.Thr68Asn alters the structure and dynamics of g-actin SD2
(A and B) Comparison of representative MD-derived snapshots of WT (A) and p.Thr68Asn (B) g-actin highlights the structural and dynamic changes induced by p.Thr68Asn (red ribbon). Side-chain atoms of relevant residues are shown and annotated.
(C) Bar heights correspond to the fraction of time that select residue pairs spent in contact with one another, averaged over triplicate simulations (error bars correspond to SD). p.Thr68Asn (orange bars) led to shifts in several amino acid interactions relative to the WT (black bars). Statistically significant differences between the WT and p.Thr68Asn contact frequencies are denoted (ns, not significant; $*p \leq 0.05$, $**p \leq 0.01$).
(D) p.Thr68Asn (orange) increased the α helix secondary structure content of residues 40–50 within the D-loop relative to the WT (black).
(E) The p.Thr68Asn variant (orange) led to a decrease in the SASA of D-loop residues (41–56) relative to the WT (black). The histogram shows the SASA probability density of all replicate simulations combined.

through SD4 and/or SD2 before ultimately altering SD2 structure (Figure 5; supplemental information; Table S2). Although operating through distinct mechanisms, all *ACTC1* variants simulated in this study led to a common change in the structure and dynamics of SD2 and the D-loop.

p.Thr68Asn modified interactions between actin subunits in F-actin

In our g-actin simulations, all variants led to similar structural changes in SD2 and the D-loop. To make predictions about the effects of altered SD2 structure/dynamics in actin filaments, we performed MD simulations of WT and p.Thr68Asn cardiac F-actin pentamers (Figure 3B). Simulation performance rapidly decreases with the number of atoms; therefore, we simulated pentamers as a simplified proxy for actin filaments and only analyzed the dynamics of the central chain (chain C) to avoid end effects. F-actin filaments are composed of two protofibrils, each of which contains monomers arranged so that SD3 of one monomer is inserted into the cleft between SD2 and SD4 of the succeeding monomer (moving from the – to + end). Two protofilaments twist around one another, and the face of the monomer containing the variant (the front-facing side in Figure 3B) is buried. SD2 is a critical structural component of actin filaments; it forms interactions between actins within a single protofilament (intra-filament) and between monomers of different protofilaments (inter-filament). For example, in the cryoelectron microscopy (cryo-EM) structure, the D-loop of one monomer encircles Y171 of the succeeding monomer in the same protofilament (in our model, the D-loop of chain C loops around Y171 of chain A). Additionally, R41 forms a salt bridge with E272 of a monomer in the opposite protofilament. In GaMD simulations of F-actin, p.Thr68Asn resulted in a shift in the structure and dynamics of SD2 and the D-loop (Figure 6). The variant resulted in a change in residue-residue interactions made by the D-loop (Figure 6A) and reduced the number of contacts made between SD2 of chain C and atoms in other actin subunits (Figure 6B). As observed in the g-actin simulations, p.Thr68Asn promoted a more compact conformation of the D-loop in F-actin (Figure 6C). The more compact loop conformation reduced interactions between the D-loop of chain C and Y171 of chain A and also eliminated the salt bridge formed between R41 of chain C and E272 of chain B (Figures 6D and 6E).

Discussion

We identified five unrelated families in which a total of eight individuals have heterozygous, rare, pathogenic variants in *ACTC1* and share similar phenotypic effects, including multiple congenital contractures, neck pterygia, scoliosis, and congenital heart defects/cardiomyopathy. This pattern of clinical findings appears to represent an autosomal dominant disorder, distinct from previously reported Mendelian conditions due to *ACTC1* variants which are characterized

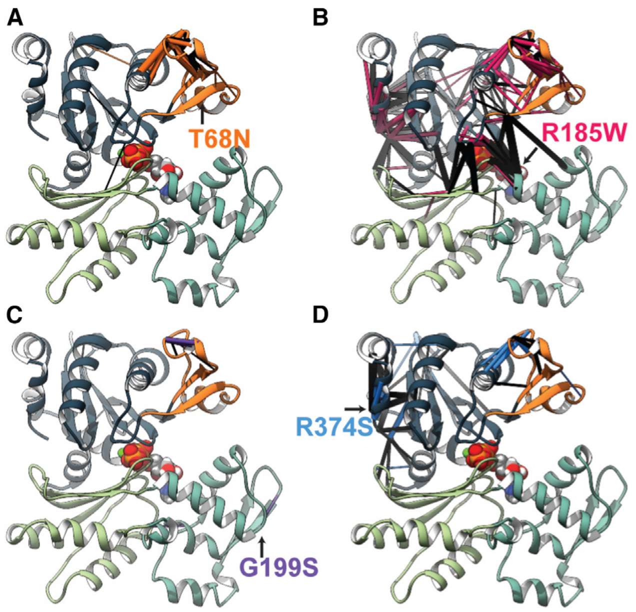

Figure 5. DA-associated variants lead to structural changes within SD2
(A–D) The percent simulation time for which residue-residue contacts endured were compared between the four mutant simulations (p.Thr68Asn [A; orange], p.Arg185Trp [B; magenta], p.Gly199Ser [C; purple], and p.Arg374Ser [D; blue]), and the WT simulations (black). For each mutant-WT comparison, residue-residue contacts that were present for statistically different percentages of the simulations were mapped onto the reference crystal structure of g-actin. Contacts that were present more frequently in the WT simulations are denoted by black pipes, and contacts present more frequently in the mutant simulations are colored orange, magenta, purple, or blue. The thickness of the pipes corresponds to the difference in percent simulation time that the contact was present between the WT and mutant simulations (larger pipes indicate that a contact was observed more frequently). Although the variants were distributed throughout the structure, they all led to statistically significant (see Table S2 for test statistics) changes in the structure of SD2 (orange ribbons).

by cardiac abnormalities, including autosomal dominant atrial septal defects [MIM: 612794],[44] dilated cardiomyopathy [MIM: 613424],[45] hypertrophic cardiomyopathy [MIM: 612098],[46] and left ventricular noncompaction [MIM: 613424][47]). MD simulations demonstrate that all four variants (p.Thr68Asn, p.Arg185Trp, G19S, and p.Arg374Ser) disrupt the native structure of the regions of actin most associated with protein-protein interactions (SD2 and the D-loop), impeding interactions between actin and its binding partners, including other actins, during thin filament assembly. Additionally, the altered D-loop structure is predicted to increase structural disorder within thin filaments, resulting in "stretchier" thin filaments that may contract more slowly, require greater loads to extend, have weakened force production, and/or have slower rates of force production. Structural perturbations to the D-loop are known to affect thin filament stiffness.[48,49] Thus, while the genetic basis of this DA condition is unique compared with other DAs, the underlying mechanisms may be similar if not identical.[9,15,50–52]

All of the pathogenic *ACTC1* variants (n = 87) reported to date (Table S1), with the exception of p.Arg185Trp, which we also identified in family D, were found in persons noted only to have abnormalities of the heart. While it is possible that congenital contractures have been overlooked in thoe families, this seems like an unlikely explanation for all or even most families. Alternatively, there may be biological explanations for this observation, none of which are mutually exclusive. First, none of the residues perturbed in the families we identified, except for Arg185, which has been found previously in a person with congenital contractures, have been reported previously. So, the distribution of phenotypic effects associated with these genotypes has been, to date, unknown. Second, substitutions of each of these residues in ACTC1 increases disorder of actin SD2 and D-loop interactions, and these perturbations could be a unique consequence of contracture-associated variants. Third, the presence of a pathogenic *ACTC1* variant may be necessary but not sufficient for development of congenital contractures. In other words, skeletal muscle might be affected only in the presence of (a) genetic modifier(s). We verified the absence of additional rare coding *ACTC1* or *ACTA1* variants but could not exclude the presence of structural variants and/or variants in non-coding regulatory elements that might alter expression of *ACTC1* or *ACTA1*.

The observation that rare genotypes in *ACTC1* underlie cardiac and skeletal abnormalities is not without precedent. ACTC1 and ACTA1 are highly homologous, differing by only four amino acids (Figure 2), and both actins are expressed in skeletal and cardiac muscle.[53–56] During fetal development, ACTC1 is the predominant actin, as measured by protein expression, in skeletal and cardiac muscle.[55] It is downregulated starting around 27–28 weeks of fetal development and continues to decline until ~6 months of age, when it accounts for ~5% of total actin.[55] In adult skeletal muscle, ACTC1 and ACTA1 account for ~5% and 95% of actin, respectively,[55] and ACTC1 accounts for ~80% of actin in adult cardiac muscle.[53] These differences in spatial and temporal expression are considered explanations for the exclusive association of skeletal muscle abnormalities (i.e., nemaline myopathy [MIM: 161800], actin accumulation myopathy [MIM: 161800], congenital fiber-type disproportion [MIM: 255310], intranuclear rod myopathy [MIM: 161800], etc.) with pathogenic *ACTA1* variants and cardiac abnormalities (autosomal dominant atrial septal defects [MIM: 612794],[44] dilated [MIM: 613424][45] and hypertrophic [MIM: 612098][46] cardiomyopathy, and left ventricular noncompaction [MIM: 613424][47]) with pathogenic variants in *ACTC1*. Of the hundreds of individuals described with *ACTA1*-associated myopathy, only 12 (9 unique variants)[57–65] have been reported to also have a cardiac abnormality (Table S1), either in conjunction with a skeletal myopathy (n = 10) or alone (n = 2). Rare variants in *ACTC1* resulting in congenital contractures in a small fraction of persons with *ACTC1* variants appears to be the corollary.

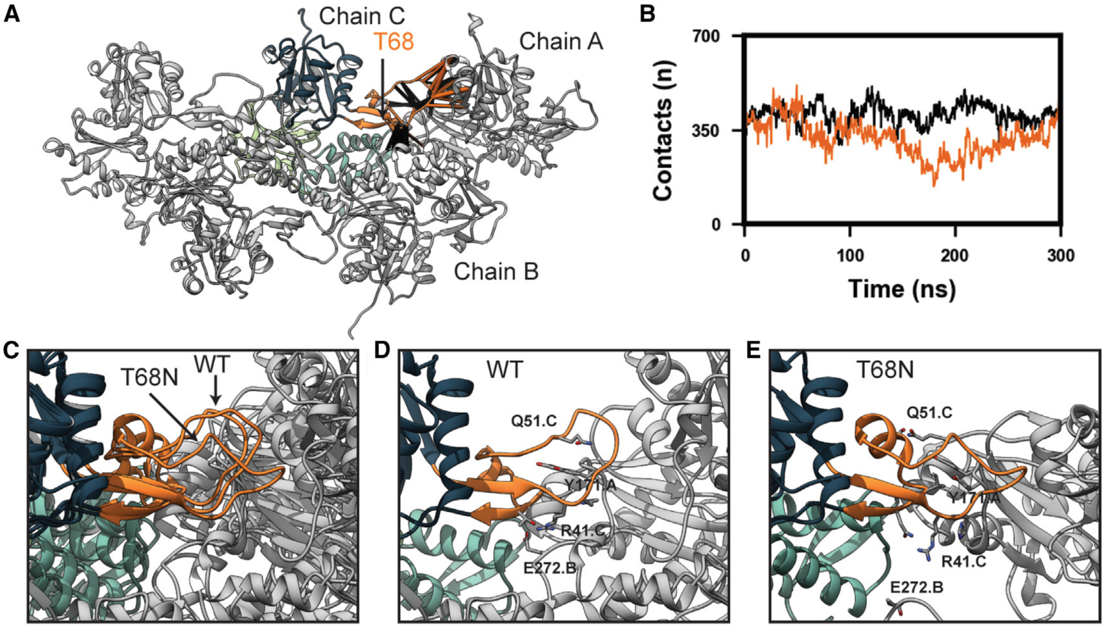

Figure 6. p.Thr68Asn alters inter-chain interactions made by SD2 in F-actin
Residue-residue interactions formed between SD2 of chain C and chains A and B were analyzed in the GaMD simulations of the WT and p.Thr68Asn F-actin.
(A) p.Thr68Asn led to statistically significant differences in residue-residue contacts formed by SD2 of chain C (denoted by pipes as in Figure 5). Differences were found in contacts formed between SD2 of chain C and SD1 of chain A as well as in contacts formed between SD2 of chain C and the SD3-SD4 linker of chain B.
(B) The total number of atom-atom interactions formed between SD2 of chain C and all atoms in chains A and B were monitored in the WT and p.Thr68Asn GaMD simulations. Relative to the WT simulation (black), the p.Thr68Asn simulation (orange) had fewer inter-chain contacts involving chain CSD2.
(C–E) In the reference cryo-EM structure and WT simulation, the D-loop of SD2 in chain C fits into a pocket formed by SD1 and SD3 of chain A. The p.Thr68Asn simulations instead sampled non-native conformations in which the D-loop exited this binding pocket. In the reference cryo-EM structure and the WT simulation (D), the D-loop of chain C is stabilized via a network of hydrophobic interactions formed with Y171 of chain A as well as a hydrogen bond network involving Arg 41 (chain C), Thr 68 (chain C), and Glu 272 (chain B). These interactions were disrupted in the p.Thr68Asn simulation (E).

Pathogenic variants in *ACTC1* result in skeletal muscle contractures even though ACTC1 accounts for only ~5% of total actin in adult skeletal muscle.[53] The most likely explanation is that the skeletal muscle contractures originate during fetal development, when ACTC1 is the predominant source of sarcomeric actin, and replacement of most skeletal muscle actin with wildtype ACTA1 during infancy is insufficient to correct the abnormality. However, it is possible that *ACTC1* has a previously unknown function in skeletal muscle biology or that mutant ACTC1 protein interferes with the function of actin encoded by *ACTA1*. Testing this hypothesis will require further functional characterization of these variants.

The MD simulations are limited by several factors. First and foremost, the method used to introduce the pathogenic variants assumes that the mutant constructs can access WT-like conformations, and the timescale along which they transition from a WT-like ensemble to a mutant ensemble is not known. Second, our simulations have probed isolated states of g-actin and F-actin and cannot directly describe effects the variants have on interactions between g-actin and its binding partners nor between F-actin and the rest of the contractile machinery present in sarcomeres. Nevertheless, these simulations provide predictions about the functional consequences of DA-associated variants in *ACTC1*, generate hypotheses on disease mechanisms, and provide guidance for future studies, such as investigation of whether the variants result in impaired filament assembly or impaired filament mechanics.

In summary, we identified five unrelated families with heterozygous pathogenic variants in *ACTC1* resulting in multiple congenital contractures, webbed neck, scoliosis, short stature, and distinctive facial features as well as cardiac abnormalities, including atrial and ventricular septal defects, left ventricular noncompaction, and cardiomyopathy. This appears to be a novel Mendelian condition because of pathogenic variants in a gene known to underlie conditions characterized only by cardiac defects. Our findings suggest that persons with multiple congenital contractures should be tested for pathogenic variants in *ACTC1* and that persons with contractures and pathogenic variants in *ACTC1* should undergo cardiac evaluation for structural and functional abnormalities.

Data and code availability

Sequence data for family A is in dbGaP under accession number phs000693, and those for families B and C will be available in

the AnVIL under accession number phs003047 pending the first public release of the GREGoR dataset. Please contact the corresponding author, M.J.B., for further information.

Supplemental information

Supplemental information can be found online at https://doi.org/10.1016/j.xhgg.2023.100213.

Acknowledgments

We thank the families for their participation and support. Sequencing and data analysis were provided by the University of Washington Center for Rare Disease Research (UW-CRDR) with support from NHGRI grants U01 HG011744, UM1 HG006493, and U24 HG011746. Protein simulations were performed with resources provided by the UW Center for Translational Muscle Research (PI, Regnier), which is supported by the National Institute of Arthritis and Musculoskeletal and Skin Diseases of the National Institutes of Health under award P30AR074990. Additional funding support for M.R. and M.C.C. was provided by NIH RM1 GM131981. This work was also funded by the National Institute of Child Health and Human Development (4R01HD048895 to M.J.B.) and a Cardiovascular Research Training Grant from the National Heart, Lung, and Blood Institute (T32HL007828 to M.C.C.). The content is solely the responsibility of the authors and does not necessarily represent the official views of the National Institutes of Health.

Declaration of interests

M.J.B. and J.X.C. are the Editor-in-Chief and Deputy Editor of *HGG Advances*, respectively, and were recused from the editorial handling of this manuscript.

Received: March 6, 2023
Accepted: June 12, 2023

Web resources

Geno2MP, https://geno2mp.gs.washington.edu/Geno2MP/.

gnomAD, http://gnomad.broadinstitute.org.

Human Genome Variation, http://www.hgvs.org/mutnomen/.

MyGene2, https://mygene2.org.

Online Mendelian Inheritance in Man (OMIM), http://www.omim.org/.

References

1. Dowling, J.J., Weihl, C.C., and Spencer, M.J. (2021). Molecular and cellular basis of genetically inherited skeletal muscle disorders. Nat. Rev. Mol. Cell Biol. *22*, 713–732. https://doi.org/10.1038/s41580-021-00389-z.
2. Au, Y. (2004). The muscle ultrastructure: a structural perspective of the sarcomere. Cell. Mol. Life Sci. *61*, 3016–3033. https://doi.org/10.1007/s00018-004-4282-x.
3. Lindskog, C., Linné, J., Fagerberg, L., Hallström, B.M., Sundberg, C.J., Lindholm, M., Huss, M., Kampf, C., Choi, H., Liem, D.A., et al. (2015). The human cardiac and skeletal muscle proteomes defined by transcriptomics and antibody-based profiling. BMC Genom. *16*, 475. https://doi.org/10.1186/s12864-015-1686-y.
4. Sung, S.S., Brassington, A.-M.E., Grannatt, K., Rutherford, A., Whitby, F.G., Krakowiak, P.A., Jorde, L.B., Carey, J.C., and Bamshad, M. (2003). Mutations in Genes Encoding Fast-Twitch Contractile Proteins Cause Distal Arthrogryposis Syndromes. Am. J. Hum. Genet. *72*, 681–690. https://doi.org/10.1086/368294.
5. Sung, S.S., Brassington, A.-M.E., Krakowiak, P.A., Carey, J.C., Jorde, L.B., and Bamshad, M. (2003). Mutations in TNNT3 cause multiple congenital contractures: a second locus for distal arthrogryposis type 2B. Am. J. Hum. Genet. *73*, 212–214. https://doi.org/10.1086/376418.
6. Toydemir, R.M., Rutherford, A., Whitby, F.G., Jorde, L.B., Carey, J.C., and Bamshad, M.J. (2006). Mutations in embryonic myosin heavy chain (MYH3) cause Freeman-Sheldon syndrome and Sheldon-Hall syndrome. Nat. Genet. *38*, 561–565. https://doi.org/10.1038/ng1775.
7. Toydemir, R.M., Chen, H., Proud, V.K., Martin, R., van Bokhoven, H., Hamel, B.C.J., Tuerlings, J.H., Stratakis, C.A., Jorde, L.B., and Bamshad, M.J. (2006). Trismus-pseudocamptodactyly syndrome is caused by recurrent mutation of MYH8. Am. J. Med. Genet. *140*, 2387–2393. https://doi.org/10.1002/ajmg.a.31495.
8. Bamshad, M., Jorde, L.B., and Carey, J.C. (1996). A revised and extended classification of the distal arthrogryposes. Am. J. Med. Genet. *65*, 277–281. https://doi.org/10.1002/(sici)1096-8628(19961111)65:4<277::aid-ajmg6>3.0.co;2-m.
9. Racca, A.W., Beck, A.E., McMillin, M.J., Korte, F.S., Bamshad, M.J., and Regnier, M. (2015). The embryonic myosin R672C mutation that underlies Freeman-Sheldon syndrome impairs cross-bridge detachment and cycling in adult skeletal muscle. Hum. Mol. Genet. *24*, 3348–3358. https://doi.org/10.1093/hmg/ddv084.
10. Mcmillin, M.J., Beck, A.E., Chong, J.X., Shively, K.M., Buckingham, K.J., Gildersleeve, H.I.S., Aracena, M.I., Aylsworth, A.S., Bitoun, P., Carey, J.C., et al. (2014). Mutations in PIEZO2 cause Gordon syndrome, Marden-Walker syndrome, and distal arthrogryposis type 5. Am. J. Hum. Genet. *94*, 734–744. https://doi.org/10.1016/j.ajhg.2014.03.015.
11. Coste, B., Houge, G., Murray, M.F., Stitziel, N., Bandell, M., Giovanni, M.A., Philippakis, A., Hoischen, A., Riemer, G., Steen, U., et al. (2013). Gain-of-function mutations in the mechanically activated ion channel PIEZO2 cause a subtype of Distal Arthrogryposis. Proc. Natl. Acad. Sci. USA *110*, 4667–4672. https://doi.org/10.1073/pnas.1221400110.
12. Mcmillin, M.J., Below, J.E., Shively, K.M., Beck, A.E., Gildersleeve, H.I., Pinner, J., Gogola, G.R., Hecht, J.T., Grange, D.K., Harris, D.J., et al. (2013). Mutations in ECEL1 Cause Distal Arthrogryposis Type 5D. Am. J. Hum. Genet. *92*, 150–156. https://doi.org/10.1016/j.ajhg.2012.11.014.
13. Dieterich, K., Quijano-Roy, S., Monnier, N., Zhou, J., Fauré, J., Smirnow, D.A., Carlier, R., Laroche, C., Marcorelles, P., Mercier, S., et al. (2013). The neuronal endopeptidase ECEL1 is associated with a distinct form of recessive distal arthrogryposis. Hum. Mol. Genet. *22*, 1483–1492. https://doi.org/10.1093/hmg/dds514.
14. Putnam, E.A., Zhang, H., Ramirez, F., and Milewicz, D.M. (1995). Fibrillin–2 (FBN2) mutations result in the Marfan–like disorder, congenital contractural arachnodactyly. Nat. Genet. *11*, 456–458. https://doi.org/10.1038/ng1295-456.
15. Chong, J.X., Talbot, J.C., Teets, E.M., Previs, S., Martin, B.L., Shively, K.M., Marvin, C.T., Aylsworth, A.S., Saadeh-Haddad, R., Schatz, U.A., et al. (2020). Mutations in MYLPF Cause a

Novel Segmental Amyoplasia that Manifests as Distal Arthrogryposis. Am. J. Hum. Genet. *107*, 293–310. https://doi.org/10.1016/j.ajhg.2020.06.014.

16. McLaren, W., Gil, L., Hunt, S.E., Riat, H.S., Ritchie, G.R.S., Thormann, A., Flicek, P., and Cunningham, F. (2016). The Ensembl Variant Effect Predictor. Genome Biol. *17*, 122. https://doi.org/10.1186/s13059-016-0974-4.
17. Paila, U., Chapman, B.A., Kirchner, R., and Quinlan, A.R. (2013). GEMINI: integrative exploration of genetic variation and genome annotations. PLoS Comput. Biol. *9*, e1003153. https://doi.org/10.1371/journal.pcbi.1003153.
18. Karczewski, K.J., Francioli, L.C., Tiao, G., Cummings, B.B., Alföldi, J., Wang, Q., Collins, R.L., Laricchia, K.M., Ganna, A., Birnbaum, D.P., et al. (2020). The mutational constraint spectrum quantified from variation in 141,456 humans. Nature *581*, 434–443. https://doi.org/10.1038/s41586-020-2308-7.
19. Berman, H.M., Westbrook, J., Feng, Z., Gilliland, G., Bhat, T.N., Weissig, H., Shindyalov, I.N., and Bourne, P.E. (2000). The Protein Data Bank. Nucleic Acids Res. *28*, 235–242. https://doi.org/10.1093/nar/28.1.235.
20. The UniProt Consortium, Martin, M.J., O'Donovan, C., Magrane, M., Alpi, E., Antunes, R., Bely, B., Bingley, M., Bonilla, C., Britto, R., et al. (2017). UniProt: the universal protein knowledgebase. Nucleic Acids Res. *45*, D158–D169. https://doi.org/10.1093/nar/gkw1099.
21. Sievers, F., Wilm, A., Dineen, D., Gibson, T.J., Karplus, K., Li, W., Lopez, R., McWilliam, H., Remmert, M., Söding, J., et al. (2011). Fast, scalable generation of high-quality protein multiple sequence alignments using Clustal Omega. Mol. Syst. Biol. *7*, 539. https://doi.org/10.1038/msb.2011.75.
22. Fiser, A., and Šali, A. (2003). Modeller: Generation and Refinement of Homology-Based Protein Structure Models. Methods Enzymol. *374*, 461–491. https://doi.org/10.1016/s0076-6879(03)74020-8.
23. von der Ecken, J., Müller, M., Lehman, W., Manstein, D.J., Penczek, P.A., and Raunser, S. (2015). Structure of the F-actin–tropomyosin complex. Nature *519*, 114–117. https://doi.org/10.1038/nature14033.
24. Case, D.A., Aktulga, H.M., Belfon, K., Ben-Shalom, I.Y., Brozell, S.R., Cerutti, D.S., Cheatham, T.E., III, Cisneros, G.A., Cruzeiro, V.W.D., et al. (2021). Amber 2021. https://ambermd.org/doc12/Amber21.pdf.
25. Case, D.A., Cheatham, T.E., Darden, T., Gohlke, H., Luo, R., Merz, K.M., Onufriev, A., Simmerling, C., Wang, B., and Woods, R.J. (2005). The Amber biomolecular simulation programs. J. Comput. Chem. *26*, 1668–1688. https://doi.org/10.1002/jcc.20290.
26. Maier, J.A., Martinez, C., Kasavajhala, K., Wickstrom, L., Hauser, K.E., and Simmerling, C. (2015). ff14SB: Improving the Accuracy of Protein Side Chain and Backbone Parameters from ff99SB. J. Chem. Theor. Comput. *11*, 3696–3713. https://doi.org/10.1021/acs.jctc.5b00255.
27. Jorgensen, W.L., Chandrasekhar, J., Madura, J.D., Impey, R.W., and Klein, M.L. (1983). Comparison of simple potential functions for simulating liquid water. J. Chem. Phys. *79*, 926–935. https://doi.org/10.1063/1.445869.
28. Li, P., Song, L.F., and Merz, K.M. (2015). Systematic Parameterization of Monovalent Ions Employing the Nonbonded Model. J. Chem. Theor. Comput. *11*, 1645–1657. https://doi.org/10.1021/ct500918t.
29. Li, P., Song, L.F., and Merz, K.M. (2015). Parameterization of Highly Charged Metal Ions Using the 12-6-4 LJ-Type Nonbonded Model in Explicit Water. J. Phys. Chem. B *119*, 883–895. https://doi.org/10.1021/jp505875v.
30. Li, P., and Merz, K.M. (2014). Taking into Account the Ion-Induced Dipole Interaction in the Nonbonded Model of Ions. J. Chem. Theor. Comput. *10*, 289–297. https://doi.org/10.1021/ct400751u.
31. Meagher, K.L., Redman, L.T., and Carlson, H.A. (2003). Development of polyphosphate parameters for use with the AMBER force field. J. Comput. Chem. *24*, 1016–1025. https://doi.org/10.1002/jcc.10262.
32. Miao, Y., Feher, V.A., and McCammon, J.A. (2015). Gaussian Accelerated Molecular Dynamics: Unconstrained Enhanced Sampling and Free Energy Calculation. J. Chem. Theor. Comput. *11*, 3584–3595. https://doi.org/10.1021/acs.jctc.5b00436.
33. Roe, D.R., and Cheatham, T.E. (2013). PTRAJ and CPPTRAJ: Software for Processing and Analysis of Molecular Dynamics Trajectory Data. J. Chem. Theor. Comput. *9*, 3084–3095. https://doi.org/10.1021/ct400341p.
34. Pettersen, E.F., Goddard, T.D., Huang, C.C., Couch, G.S., Greenblatt, D.M., Meng, E.C., and Ferrin, T.E. (2004). UCSF Chimera–a visualization system for exploratory research and analysis. J. Comput. Chem. *25*, 1605–1612. https://doi.org/10.1002/jcc.20084.
35. Sanner, M.F., Olson, A.J., and Spehner, J.C. (1996). Reduced surface: An efficient way to compute molecular surfaces. Biopolymers *38*, 305–320. https://doi.org/10.1002/(sici)1097-0282(199603)38:3<305::aid-bip4>3.0.co;2-y.
36. Chong, J.X., Burrage, L.C., Beck, A.E., Marvin, C.T., McMillin, M.J., Shively, K.M., Harrell, T.M., Buckingham, K.J., Bacino, C.A., Jain, M., et al. (2015). Autosomal-Dominant Multiple Pterygium Syndrome Is Caused by Mutations in MYH3. Am. J. Hum. Genet. *96*, 841–849. https://doi.org/10.1016/j.ajhg.2015.04.004.
37. Rentzsch, P., Schubach, M., Shendure, J., and Kircher, M. (2021). CADD-Splice—improving genome-wide variant effect prediction using deep learning-derived splice scores. Genome Med. *13*, 31. https://doi.org/10.1186/s13073-021-00835-9.
38. Agrawal, P.B., Strickland, C.D., Midgett, C., Morales, A., Newburger, D.E., Poulos, M.A., Tomczak, K.K., Ryan, M.M., Iannaccone, S.T., Crawford, T.O., et al. (2004). Heterogeneity of nemaline myopathy cases with skeletal muscle alpha-actin gene mutations. Ann. Neurol. *56*, 86–96. https://doi.org/10.1002/ana.20157.
39. Laing, N.G., Dye, D.E., Wallgren-Pettersson, C., Richard, G., Monnier, N., Lillis, S., Winder, T.L., Lochmüller, H., Graziano, C., Mitrani-Rosenbaum, S., et al. (2009). Mutations and polymorphisms of the skeletal muscle alpha-actin gene (ACTA1). Hum. Mutat. *30*, 1267–1277. https://doi.org/10.1002/humu.21059.
40. Sparrow, J.C., Nowak, K.J., Durling, H.J., Beggs, A.H., Wallgren-Pettersson, C., Romero, N., Nonaka, I., and Laing, N.G. (2003). Muscle disease caused by mutations in the skeletal muscle alpha-actin gene (ACTA1). Neuromuscul. Disord. *13*, 519–531. https://doi.org/10.1016/s0960-8966(03)00101-9.
41. Ilkovski, B., Nowak, K.J., Domazetovska, A., Maxwell, A.L., Clement, S., Davies, K.E., Laing, N.G., North, K.N., and Cooper, S.T. (2004). Evidence for a dominant-negative effect in ACTA1 nemaline myopathy caused by abnormal folding, aggregation and altered polymerization of mutant actin isoforms. Hum. Mol. Genet. *13*, 1727–1743. https://doi.org/10.1093/hmg/ddh185.
42. Ilkovski, B., Cooper, S.T., Nowak, K., Ryan, M.M., Yang, N., Schnell, C., Durling, H.J., Roddick, L.G., Wilkinson, I., Kornberg, A.J., et al. (2001). Nemaline myopathy caused by

mutations in the muscle alpha-skeletal-actin gene. Am. J. Hum. Genet. *68*, 1333–1343. https://doi.org/10.1086/320605.
43. Nowak, K.J., Wattanasirichaigoon, D., Goebel, H.H., Wilce, M., Pelin, K., Donner, K., Jacob, R.L., Hübner, C., Oexle, K., Anderson, J.R., et al. (1999). Mutations in the skeletal muscle alpha-actin gene in patients with actin myopathy and nemaline myopathy. Nat. Genet. *23*, 208–212. https://doi.org/10.1038/13837.
44. Matsson, H., Eason, J., Bookwalter, C.S., Klar, J., Gustavsson, P., Sunnegårdh, J., Enell, H., Jonzon, A., Vikkula, M., Gutierrez, I., et al. (2008). Alpha-cardiac actin mutations produce atrial septal defects. Hum. Mol. Genet. *17*, 256–265. https://doi.org/10.1093/hmg/ddm302.
45. Olson, T.M., Michels, V.V., Thibodeau, S.N., Tai, Y.-S., and Keating, M.T. (1998). Actin Mutations in Dilated Cardiomyopathy, a Heritable Form of Heart Failure. Science *280*, 750–752. https://doi.org/10.1126/science.280.5364.750.
46. Mogensen, J., Klausen, I.C., Pedersen, A.K., Egeblad, H., Bross, P., Kruse, T.A., Gregersen, N., Hansen, P.S., Baandrup, U., and Børglum, A.D. (1999). α-cardiac actin is a novel disease gene in familial hypertrophic cardiomyopathy. J. Clin. Invest. *103*, R39–R43. https://doi.org/10.1172/jci6460.
47. Monserrat, L., Hermida-Prieto, M., Fernandez, X., Rodríguez, I., Dumont, C., Cazón, L., Cuesta, M.G., Gonzalez-Juanatey, C., Peteiro, J., Alvarez, N., et al. (2007). Mutation in the alpha-cardiac actin gene associated with apical hypertrophic cardiomyopathy, left ventricular non-compaction, and septal defects. Eur. Heart J. *28*, 1953–1961. https://doi.org/10.1093/eurheartj/ehm239.
48. Hocky, G.M., Baker, J.L., Bradley, M.J., Sinitskiy, A.V., De La Cruz, E.M., and Voth, G.A. (2016). Cations Stiffen Actin Filaments by Adhering a Key Structural Element to Adjacent Subunits. J. Phys. Chem. B *120*, 4558–4567. https://doi.org/10.1021/acs.jpcb.6b02741.
49. Chan, C., Fan, J., Messer, A.E., Marston, S.B., Iwamoto, H., and Ochala, J. (2016). Myopathy-inducing mutation H40Y in ACTA1 hampers actin filament structure and function. Biochim. Biophys. Acta *1862*, 1453–1458. https://doi.org/10.1016/j.bbadis.2016.04.013.
50. Racca, A.W., Beck, A.E., Bamshad, M.J., and Regnier, M. (2014). Prolonged Relaxation Kinetics in Distal Arthrogryposis Skeletal Muscle Myofibrils with a MYH3 R672C Mutation. Biophys. J. *106*, 771a. https://doi.org/10.1016/j.bpj.2013.11.4233.
51. Robinson, P., Lipscomb, S., Preston, L.C., Altin, E., Watkins, H., Ashley, C.C., and Redwood, C.S. (2007). Mutations in fast skeletal troponin I, troponin T, and β-tropomyosin that cause distal arthrogryposis all increase contractile function. Faseb. J. *21*, 896–905. https://doi.org/10.1096/fj.06-6899com.
52. Ha, K., Buchan, J.G., Alvarado, D.M., Mccall, K., Vydyanath, A., Luther, P.K., Goldsmith, M.I., Dobbs, M.B., and Gurnett, C.A. (2013). MYBPC1 mutations impair skeletal muscle function in zebrafish models of arthrogryposis. Hum. Mol. Genet. *22*, 4967–4977. https://doi.org/10.1093/hmg/ddt344.
53. Vandekerckhove, J., Bugaisky, G., and Buckingham, M. (1986). Simultaneous expression of skeletal muscle and heart actin proteins in various striated muscle tissues and cells. A quantitative determination of the two actin isoforms. J. Biol. Chem. *261*, 1838–1843. https://doi.org/10.1016/s0021-9258(17)36017-9.
54. Tondeleir, D., Vandamme, D., Vandekerckhove, J., Ampe, C., and Lambrechts, A. (2009). Actin isoform expression patterns during mammalian development and in pathology: Insights from mouse models. Cell Motil. Cytoskelet. *66*, 798–815. https://doi.org/10.1002/cm.20350.
55. Ilkovski, B., Clement, S., Sewry, C., North, K.N., and Cooper, S.T. (2005). Defining α-skeletal and α-cardiac actin expression in human heart and skeletal muscle explains the absence of cardiac involvement in ACTA1 nemaline myopathy. Neuromuscul. Disord. *15*, 829–835. https://doi.org/10.1016/j.nmd.2005.08.004.
56. Abdul-Hussein, S., van der Ven, P.F.M., and Tajsharghi, H. (2012). Expression profiles of muscle disease-associated genes and their isoforms during differentiation of cultured human skeletal muscle cells. Bmc Musculoskelet Di *13*, 262. https://doi.org/10.1186/1471-2474-13-262.
57. Matsumoto, A., Tsuda, H., Furui, S., Kawada-Nagashima, M., Anzai, T., Seki, M., Watanabe, K., Muramatsu, K., Osaka, H., Iwamoto, S., et al. (2022). A case of congenital fiber-type disproportion syndrome presenting dilated cardiomyopathy with ACTA1 mutation. Mol. Genet. Genomic Med. *10*, e2008. https://doi.org/10.1002/mgg3.2008.
58. Tadokoro, K., Ohta, Y., Sasaki, R., Takahashi, Y., Sato, K., Shang, J., Takemoto, M., Hishikawa, N., Yamashita, T., Nakamura, K., et al. (2018). Congenital myopathy with fiber-type disproportion accompanied by dilated cardiomyopathy in a patient with a novel p.G48A ACTA1 mutation. J. Neurol. Sci. *393*, 142–144. https://doi.org/10.1016/j.jns.2018.08.015.
59. Carnevale, A., Rosas-Madrigal, S., Rosendo-Gutiérrez, R., López-Mora, E., Romero-Hidalgo, S., Avila-Vazzini, N., Jacobo--Albavera, L., Domínguez-Pérez, M., Vargas-Alarcón, G., Pérez-Villatoro, F., et al. (2020). Genomic study of dilated cardiomyopathy in a group of Mexican patients using site-directed next generation sequencing. Mol. Genet. Genomic Med. *8*, e1504. https://doi.org/10.1002/mgg3.1504.
60. Kim, S.-Y., Park, Y.-E., Kim, H.-S., Lee, C.-H., Yang, D.H., and Kim, D.-S. (2011). Nemaline myopathy and non-fatal hypertrophic cardiomyopathy caused by a novel ACTA1 E239K mutation. J. Neurol. Sci. *307*, 171–173. https://doi.org/10.1016/j.jns.2011.04.022.
61. Reza, N., Garg, A., Merrill, S.L., Chowns, J.L., Rao, S., and Owens, A.T. (2018). ACTA1 Novel Likely Pathogenic Variant in a Family With Dilated Cardiomyopathy. Circ. Genom. Precis. Med. *11*, e002243. https://doi.org/10.1161/circgen.118.002243.
62. Kaindl, A.M., Rüschendorf, F., Krause, S., Goebel, H.-H., Koehler, K., Becker, C., Pongratz, D., Müller-Höcker, J., Nürnberg, P., Stoltenburg-Didinger, G., et al. (2004). Missense mutations of ACTA1 cause dominant congenital myopathy with cores. J. Med. Genet. *41*, 842–848. https://doi.org/10.1136/jmg.2004.020271.
63. D'Amico, A., Graziano, C., Pacileo, G., Petrini, S., Nowak, K.J., Boldrini, R., Jacques, A., Feng, J.-J., Porfirio, B., Sewry, C.A., et al. (2006). Fatal hypertrophic cardiomyopathy and nemaline myopathy associated with ACTA1 K336E mutation. Neuromuscul. Disord. *16*, 548–552. https://doi.org/10.1016/j.nmd.2006.07.005.
64. Gatayama, R., Ueno, K., Nakamura, H., Yanagi, S., Ueda, H., Yamagishi, H., and Yasui, S. (2013). Nemaline Myopathy With Dilated Cardiomyopathy in Childhood. Pediatrics *131*, e1986–e1990. https://doi.org/10.1542/peds.2012-1139.
65. Yokoyama, S., Koide, A., Nishino, I., Hayashi, Y., Ohki, H., Miura, M., and Shibuya, K. (2016). Hypertrophic cardiomyopathy associated with nemaline myopathy due to ACTA1 mutation. Ped. Cardiol. Card. Surg. *32*, 181–186. https://doi.org/10.9794/jspccs.32.181.

HGGA, Volume *4*

Supplemental information

Variants in *ACTC1* underlie distal arthrogryposis accompanied by congenital heart defects

Jessica X. Chong, Matthew Carter Childers, Colby T. Marvin, Allison J. Marcello, Hernan Gonorazky, Lili-Naz Hazrati, James J. Dowling, Fatema Al Amrani, Yasemin Alanay, Yolanda Nieto, Miguel Á Marín Gabriel, Arthur S. Aylsworth, Kati J. Buckingham, Kathryn M. Shively, Olivia Sommers, Kailyn Anderson, University of Washington Center for Mendelian Genomics, University of Washington Center for Rare Disease Research, Michael Regnier, and Michael J. Bamshad

Supplemental Materials and Methods

Table of Contents

1. Summary of structural effects of R185W, G199S, and R374S
2. Figure S1. Pedigrees of families with pathogenic variants in *ACTC1* resulting in distal arthrogryposis
3. Figure S2. Histochemistry and immunohistochemistry from muscle biopsy of paravertebral muscles in proband of Family D.
4. Figure S3. Pathogenic variant-associated changes in C_α RMSF of g-actin.
5. Figure S4. Pathogenic variant-associated changes in D-loop – subdomain 2 contacts.
6. Figure S5. Pathogenic variant-associated changes in D-loop secondary structure.
7. Figure S6. Pathogenic variant-associated changes in D-loop solvent accessible surface area.
8. Table S1. *ACTC1* and *ACTA1* missense variants reported as pathogenic in individuals with phenotypes involving cardiac muscle, skeletal muscle, or both. (provided as a separate Excel spreadsheet)
9. Table S2. Reported residue-residue pair interaction frequencies that differed among the WT and mutant simulations. (provided as a separate Excel spreadsheet and in this document)

Summary of structural effects of R185W, G199S, and R374S

R185Y: In the WT simulations, R185 formed transient electrostatic interactions with S16, G17, E74, and D159. Additionally the Y71 side chain formed a large number of atom-atom contacts with R185 by stacking against its guanidino group. Due to these interactions, R185 stabilizes g-actin structure in the vicinity of the ATP pocket and forms a structural linker between all four subdomains. Mutation of R185 to Trp eliminated these stabilizing interactions; instead, W185 was primarily positioned in the cleft between SD2 and SD4 and interacted primarily with other SD4 residues. This resulted in a large-scale reorganization of inter-residue contacts spanning all 4 subdomains and an increase in the SD2-SD4 cleft distance.

G199S: In the WT simulations, G199 adopts backbone dihedral angles that are unfavorable for most amino acids and accommodates a transition from α-helix structure in residues 184-198 to coil structure in residues 199 – 204. Mutation to S199 resulted in a shift in the backbone conformation of residue 199 to angles within the lower left quadrant of Ramachandran space, a decrease in a-helix structure in residues 197-198, and a shift in the structure of the loop spanning residues 199 to 204. This change resulted in few statistically significant changes in inter-residue interactions but did modify the breathing motion between subdomains 2 and 4.

R374S: In the WT simulations, R374 formed transient salt bridges with E363 and E366. Each of these residues are located on the surface of SD1. These salt bridges were lost in the R374S simulations. Simultaneously there was a reorganization of inter-residue interactions in SD1 as well as an increase in residue-residue interactions between an α-helix of SD1 (residues 81-96) and residues in SD2.

Figure S1. Pedigrees of families with pathogenic variants in *ACTC1* resulting in distal arthrogryposis.

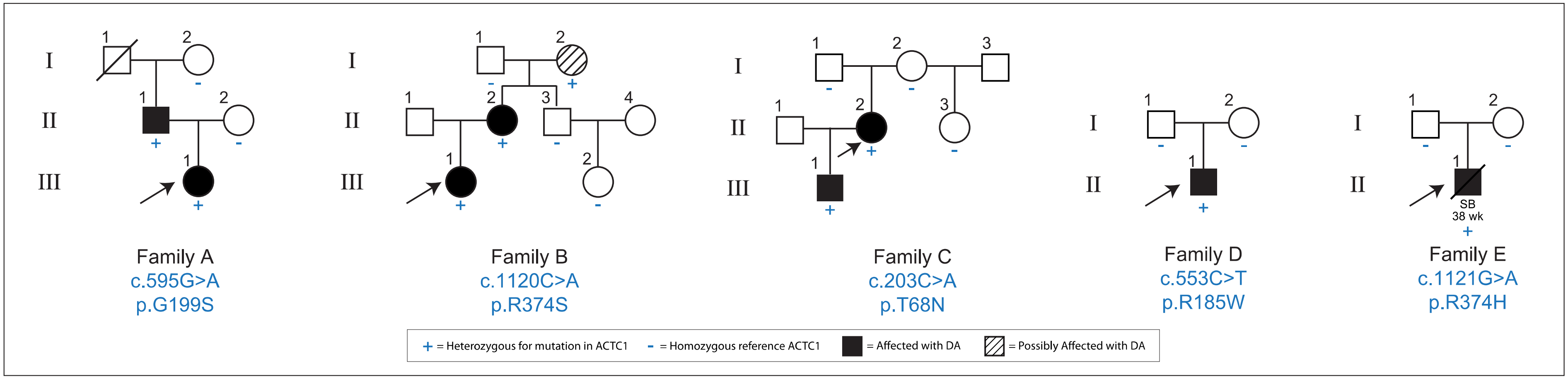

Figure S2. Histochemistry and immunohistochemistry from muscle biopsy of paravertebral muscles in proband of Family D. (A) Scattered areas of round muscle fibers found in H&E [hematoxylin and eosin] staining. (B) ATPase (pH4.2) shows type I fiber predominance that could be seen in paravertebral muscles. (C) No ragged red fibers or rod-like inclusions are seen with modified Gomori trichrome staining however scattered nemaline bodies were found in (D) electronic microscopy.

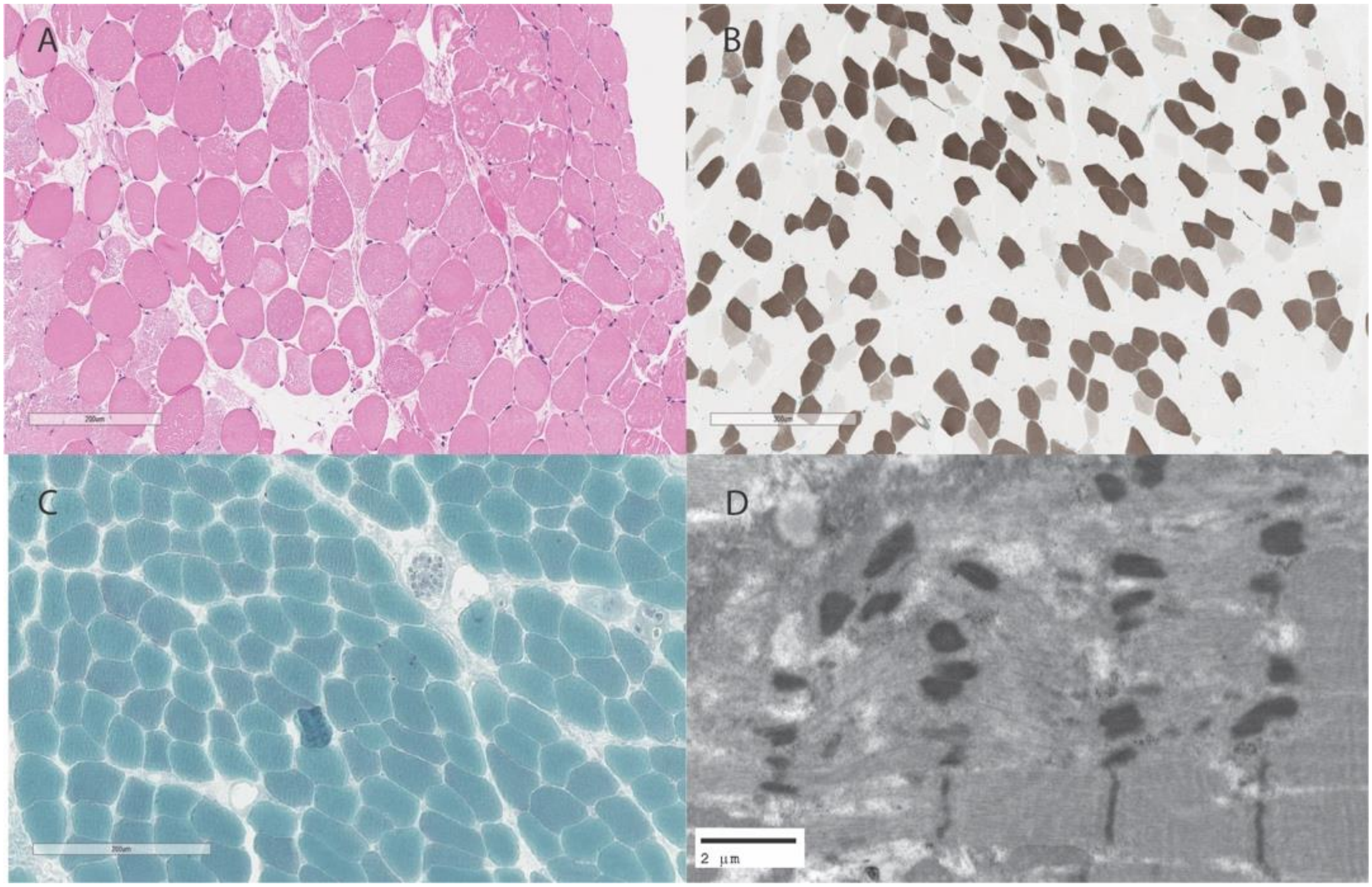

Figure S3. Pathogenic variant-associated changes in C_α RMSF of g-actin. In MD simulations, C_α RMSF measures the root-mean-squared fluctuation of the C_α atoms about their average positions. RMSF values were calculated after alignment of the g-actin trajectories to their average coordinates. Average RMSF values are reported per residue (left column) for the WT (a), T68N (b), R185W (c), G199S (d), and R374S (e)., simulations. Points correspond to the average value and shaded regions denote the standard deviation. Vertical red lines denote mutation sites. The difference in the average RMSF relative to the WT simulation of the T68N (b), R185W (c), G199S (d), and R374S (e) variants are plotted in the right column. Positive values indicate greater RMSF in WT and negative values indicate greater RMSF in the mutants. Vertical red lines denote variant sites. Red stars denote residues with statistically significant ($p < 0.05$) differences in the RMSF between the WT and mutant simulations.

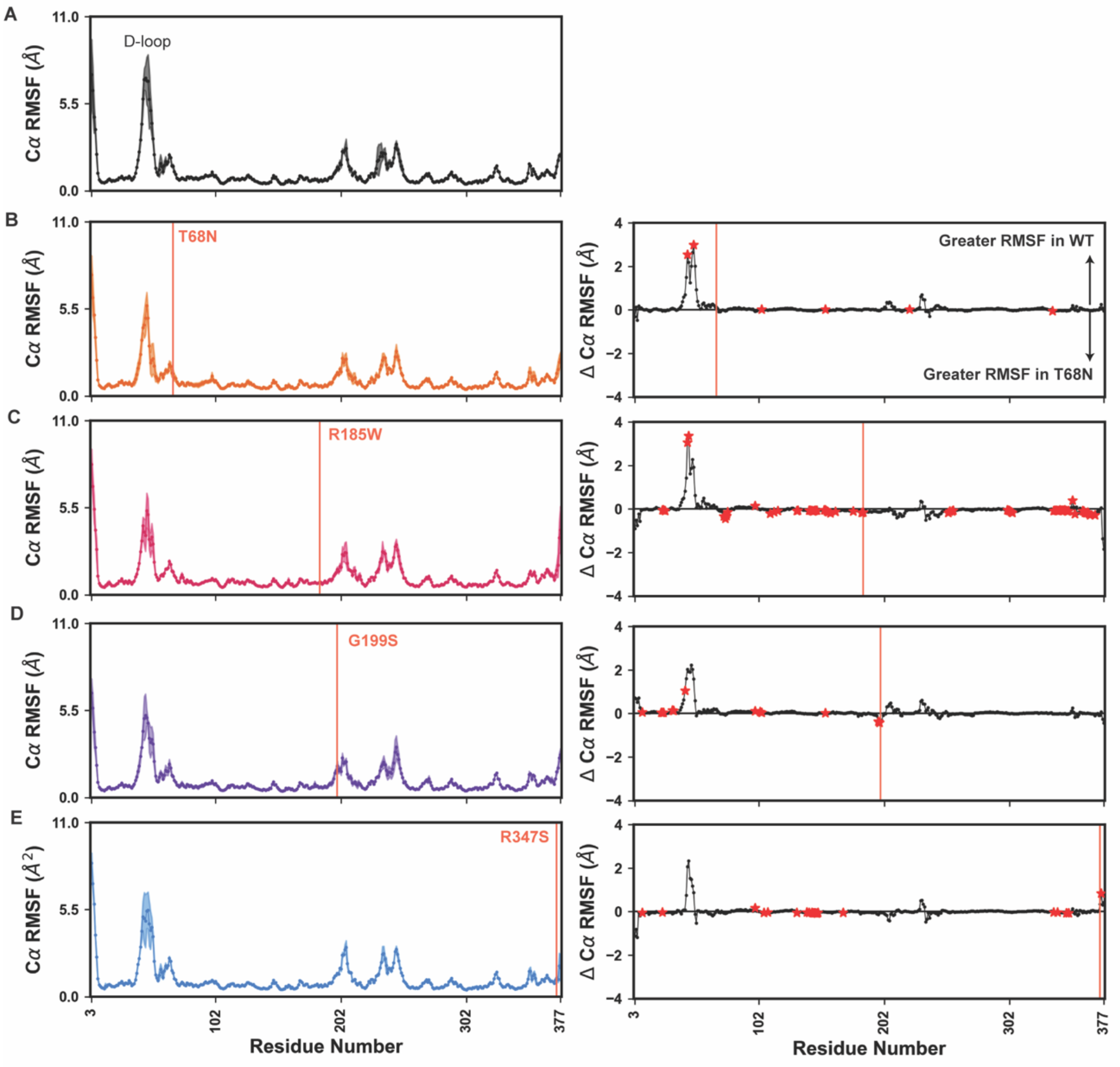

Figure S4. Pathogenic variant-associated changes in D-loop – subdomain 2 contacts. We calculated the average percent simulation time for which select residue-residue interactions were present in the WT (black), T68N (orange), R185W (magenta), G199S (purple), and R374S (blue) simulations. Error bars denote standard deviation. Statistically significant differences between the WT and T68N contact frequencies are denoted (ns: not significant, *: p ≤ 0.05, **: p ≤ 0.01)

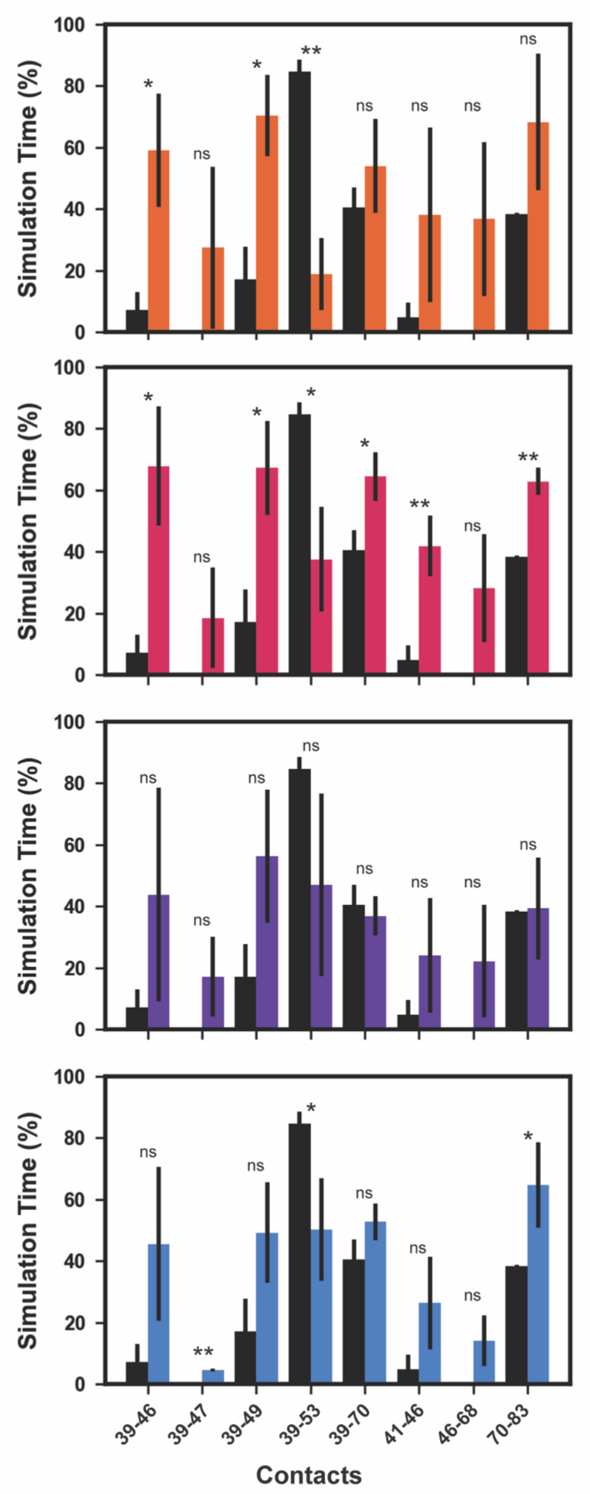

Figure S5. Pathogenic variant-associated changes in D-loop secondary structure. For each residue in the D-loop (# 40 – 56), we calculated the average percent simulation time for which the residue adopted α-helix secondary structure. The α-helix content per residue is shown for T68N (orange), R185W (magenta), G199S (purple), and R374S (blue) versus the WT (grey) simulation. All variants in this study resulted in an increase in α-helix content in the D-loop.

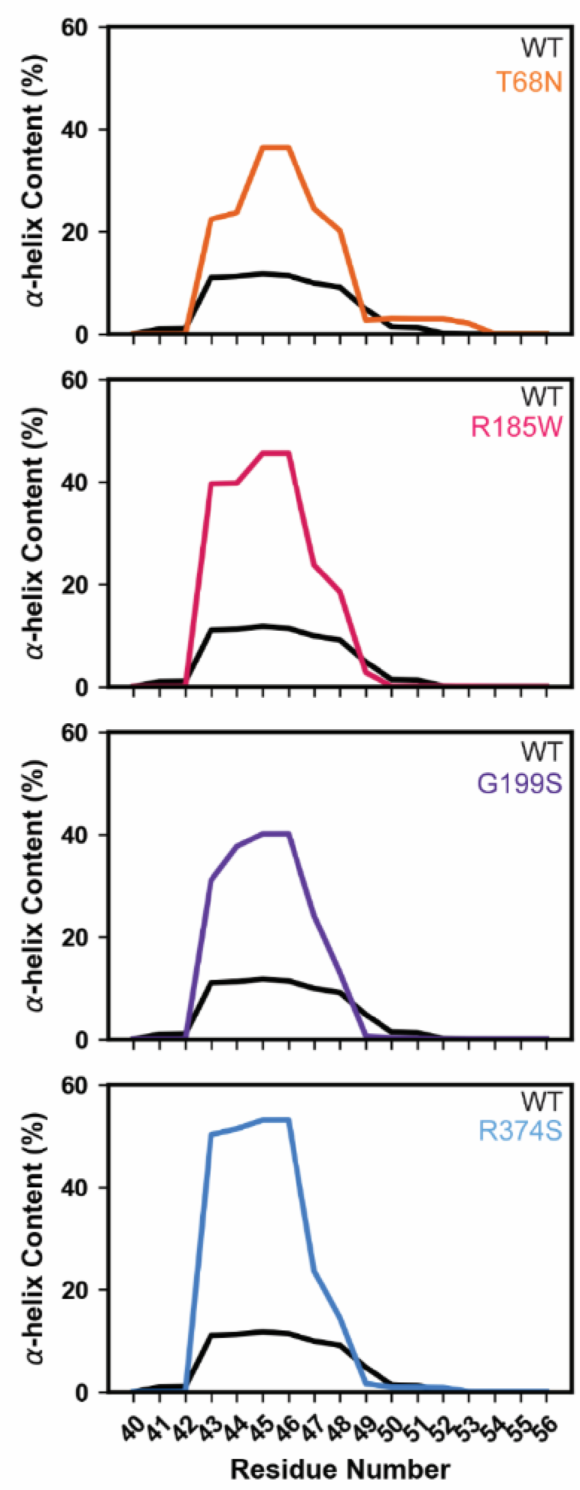

Figure S6. Pathogenic variant-associated changes in D-loop solvent accessible surface area. Histograms of the solvent accessible surface area aggregated over all replicate simulations are plotted for T68N (orange), R185W (magenta), G199S (purple), and R374S (blue) versus the WT (grey) distribution. All variants in this study resulted in a decrease in the SASA of D-loop residues.

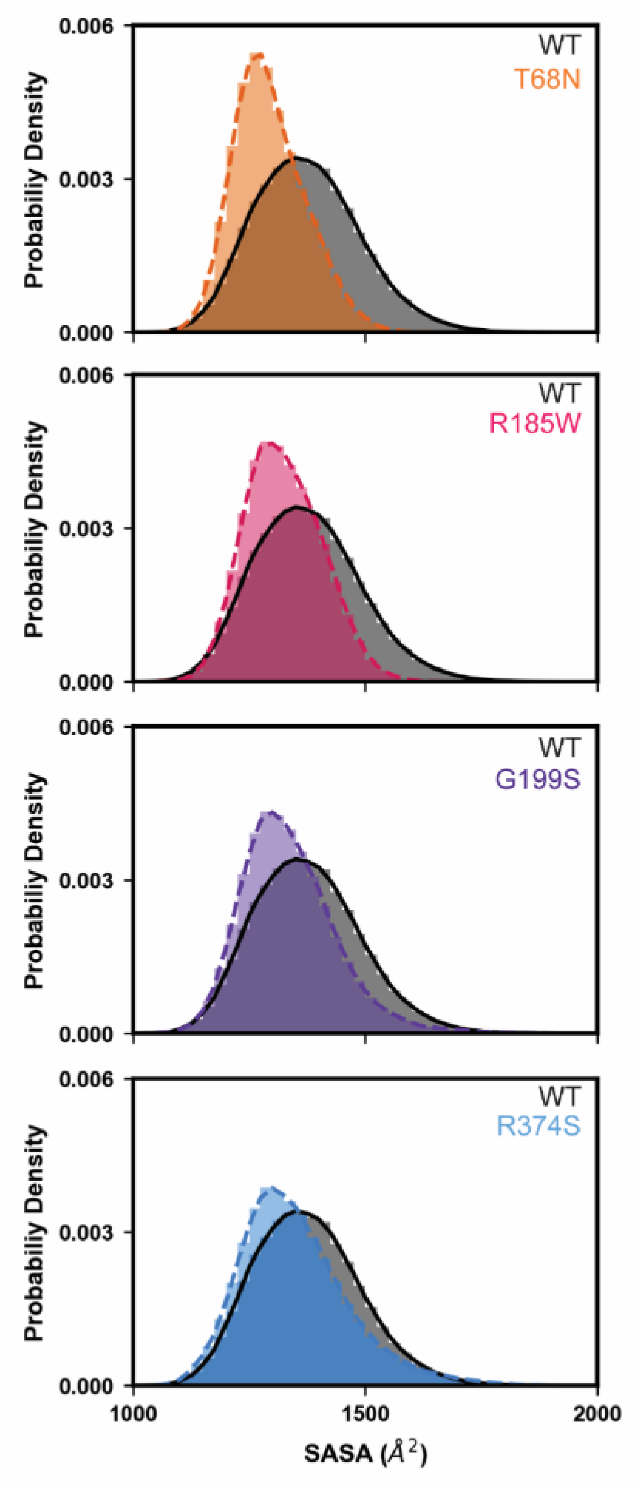

Table S1. *ACTC1* and *ACTA1* missense variants reported as pathogenic in individuals with phenotypes involving cardiac muscle, skeletal muscle, or both. This table contains all missense variants in *ACTA1* or *ACTC1* that were reported as Likely Pathogenic or Pathogenic in ClinVar; Damaging in HGMD, and/or non-synonymous in the *ACTC1* or *ACTA1* LOVD Locus Specific Databases. ACTA1 and ACTC1 are both 377 residues long and differ at only 4 sites (4, 5, 301, 360). Each variant is listed alongside the reported phenotype (one row per affected individual/database entry), whether the affected individual had skeletal muscle or cardiac muscle findings, the Pubmed PMID of any associated publication and/or submitter. The residue numbering listed for ACTA1 variants was updated to current (2022) numbering if necessary.

Table S1 is provided as an Excel spreadsheet.

Table S2. Reported residue-residue pair interaction frequencies that differed among the WT and mutant simulations. This table enumerates the average percent simulation time for which select residue-residue pairs formed inter-residue interactions in the WT and mutant MD simulations. Altered residue-residue interactions are only reported if there was at least a 10% difference in the average contact time frequency between the WT and mutant simulations and a statistically significant difference in the average WT and mutant interaction frequencies.

Table S2 is also provided as an Excel spreadsheet.

Variant	Residue I	Residue J	Contact Frequency (WT, %)	Contact Frequency (Mutant, %)	T-test Statistic	P-Value
T68N	3	103	26	4	2.834	0.047
T68N	14	121	92	80	5.711	0.005
T68N	39	46	7	59	-3.79	0.019
T68N	39	49	17	70	-4.47	0.011
T68N	39	52	42	8	3.805	0.019
T68N	39	53	85	19	7.6	0.002
T68N	40	51	33	0	3.085	0.037
T68N	41	51	13	0	5.527	0.005
T68N	41	65	74	87	-3.475	0.025
T68N	41	67	82	94	-3.185	0.033
T68N	42	51	23	0	3.551	0.024
T68N	43	51	47	3	4.354	0.012
T68N	44	51	27	0	4.167	0.014
T68N	45	49	24	76	-2.977	0.041
T68N	46	51	24	5	5.234	0.006
T68N	47	51	33	5	4.41	0.012
T68N	50	54	16	87	-5.7	0.005
T68N	51	54	13	88	-8.014	0.001
T68N	51	82	8	88	-7.869	0.001
T68N	51	83	6	59	-7.649	0.002
T68N	51	86	6	84	-7.427	0.002
T68N	52	86	22	88	-7.062	0.002
T68N	75	163	85	73	2.923	0.043
T68N	92	131	8	18	-2.846	0.047
R185W	7	355	64	18	4.728	0.009
R185W	8	355	38	2	3.4	0.027
R185W	9	355	57	4	4.689	0.009
R185W	10	92	84	96	-5.449	0.006
R185W	10	358	84	67	7.614	0.002
R185W	11	105	40	26	2.937	0.043
R185W	12	91	54	81	-6.932	0.002

**R185W**	12	92	76	50	5.995	0.004
**R185W**	12	105	56	37	4.637	0.01
**R185W**	14	19	86	72	7.18	0.002
**R185W**	14	76	8	42	-3.143	0.035
**R185W**	16	74	6	79	-5.433	0.006
**R185W**	16	75	12	85	-5.969	0.004
**R185W**	16	77	1	19	-3.258	0.031
**R185W**	16	185	100	0	726.152	0
**R185W**	17	36	25	13	3.079	0.037
**R185W**	17	185	99	0	295.7	0
**R185W**	19	34	72	85	-3.348	0.029
**R185W**	19	84	73	47	3.512	0.025
**R185W**	19	88	43	12	10.62	0
**R185W**	21	92	22	8	7.167	0.002
**R185W**	23	96	74	88	-5.732	0.005
**R185W**	23	103	73	50	3.082	0.037
**R185W**	25	347	51	39	4.542	0.01
**R185W**	31	91	40	21	5.952	0.004
**R185W**	32	58	6	27	-6.528	0.003
**R185W**	32	95	60	36	6.647	0.003
**R185W**	34	71	1	15	-7.819	0.001
**R185W**	34	87	88	68	3.949	0.017
**R185W**	35	73	63	89	-3.77	0.02
**R185W**	35	84	24	9	3.931	0.017
**R185W**	37	86	49	87	-6.088	0.004
**R185W**	37	87	87	97	-3.547	0.024
**R185W**	38	56	67	80	-3.033	0.039
**R185W**	39	46	7	68	-4.233	0.013
**R185W**	39	49	17	67	-3.824	0.019
**R185W**	39	52	42	4	5.082	0.007
**R185W**	39	53	85	38	3.823	0.019
**R185W**	39	70	40	64	-3.301	0.03
**R185W**	40	45	21	90	-4.736	0.009
**R185W**	40	46	23	91	-5.749	0.005
**R185W**	41	46	5	42	-4.787	0.009
**R185W**	41	64	50	66	-7.799	0.001
**R185W**	41	65	74	88	-3.734	0.02
**R185W**	41	67	82	95	-3.446	0.026
**R185W**	42	45	58	100	-5.57	0.005
**R185W**	42	48	14	0	3.239	0.032
**R185W**	43	51	47	9	2.922	0.043
**R185W**	44	51	27	2	3.709	0.021
**R185W**	45	49	24	74	-2.879	0.045
**R185W**	46	51	24	9	3.073	0.037
**R185W**	47	51	33	2	6.377	0.003
**R185W**	48	51	39	0	11.718	0

R185W	50	54	16	68	-3.051	0.038
R185W	51	54	13	67	-3.625	0.022
R185W	51	82	8	48	-4.509	0.011
R185W	51	85	1	30	-2.847	0.047
R185W	51	86	6	71	-4.372	0.012
R185W	52	85	2	18	-5.681	0.005
R185W	56	83	35	7	4.063	0.015
R185W	56	90	89	99	-2.79	0.049
R185W	57	90	13	37	-2.934	0.043
R185W	61	71	9	29	-7.417	0.002
R185W	70	83	38	63	-7.659	0.002
R185W	71	80	44	25	3.283	0.03
R185W	71	159	67	24	9.272	0.001
R185W	71	185	98	18	4.678	0.009
R185W	71	208	54	1	5.683	0.005
R185W	73	185	22	0	6.856	0.002
R185W	74	185	20	0	8.777	0.001
R185W	75	163	85	38	6.651	0.003
R185W	75	179	98	75	27.142	0
R185W	75	181	78	19	6.857	0.002
R185W	76	161	81	12	5.399	0.006
R185W	76	163	45	3	9.229	0.001
R185W	87	92	22	11	7.734	0.002
R185W	88	93	74	62	5.318	0.006
R185W	92	105	81	94	-5.211	0.006
R185W	93	128	50	33	3.715	0.021
R185W	100	104	39	20	5.873	0.004
R185W	100	125	19	6	24.044	0
R185W	104	355	32	3	3.012	0.039
R185W	106	136	86	60	7.426	0.002
R185W	106	137	90	72	5.361	0.006
R185W	106	348	49	87	-5.507	0.005
R185W	106	354	85	51	3.307	0.03
R185W	109	377	5	43	-2.974	0.041
R185W	110	163	78	19	4.076	0.015
R185W	118	376	0	53	-5.678	0.005
R185W	118	377	0	55	-5.725	0.005
R185W	134	357	18	52	-4.789	0.009
R185W	135	348	10	50	-7.664	0.002
R185W	135	372	56	3	2.792	0.049
R185W	135	374	61	1	3.531	0.024
R185W	136	376	1	64	-10.241	0.001
R185W	137	348	22	47	-5.38	0.006
R185W	137	376	31	1	3.251	0.031
R185W	138	377	7	47	-2.838	0.047
R185W	140	156	76	61	4.604	0.01

R185W	140	341	67	46	2.932	0.043
R185W	141	376	18	0	3.437	0.026
R185W	141	377	87	23	13.812	0
R185W	145	170	11	32	-3.201	0.033
R185W	145	377	72	16	5.403	0.006
R185W	150	377	27	4	3.163	0.034
R185W	158	302	31	6	4.475	0.011
R185W	159	186	79	30	3.04	0.038
R185W	160	185	96	22	23.03	0
R185W	160	186	70	87	-3.516	0.025
R185W	163	177	6	36	-3.618	0.022
R185W	164	299	8	25	-5.792	0.004
R185W	169	377	46	9	4.046	0.016
R185W	170	377	88	33	7.927	0.001
R185W	172	177	95	70	3.896	0.018
R185W	183	263	24	13	5.821	0.004
R185W	340	345	53	39	4.306	0.013
R185W	344	349	43	29	5.604	0.005
R185W	348	376	38	0	3.312	0.03
R185W	348	377	87	20	11.32	0
R185W	349	354	98	65	2.936	0.043
R185W	349	355	91	60	4.729	0.009
R185W	352	356	89	68	5.686	0.005
R185W	353	357	94	51	4.696	0.009
R185W	354	376	57	1	3.234	0.032
R185W	354	377	27	10	5.099	0.007
R185W	356	375	0	53	-3.947	0.017
R185W	357	374	59	3	4.13	0.014
R185W	357	376	93	26	15.169	0
R185W	358	374	59	4	4.426	0.011
R185W	358	375	0	54	-3.857	0.018
R185W	359	374	70	8	5.349	0.006
R185W	359	375	33	87	-3.402	0.027
R185W	359	376	1	53	-6.749	0.003
R185W	360	374	16	2	3.156	0.034
R185W	360	375	0	16	-3.055	0.038
R185W	363	374	79	30	5.195	0.007
R185W	363	375	0	48	-10.399	0
R185W	367	374	18	52	-3.945	0.017
R185W	370	374	42	95	-4.137	0.014
R185W	371	375	35	91	-3.582	0.023
R185W	372	376	0	67	-15.053	0
R185W	373	376	0	67	-12.103	0
R185W	373	377	0	26	-4.84	0.008
G199S	25	347	51	40	3.158	0.034
G199S	41	51	13	1	4.313	0.013

G199S	44	48	25	72	-4.363	0.012
G199S	45	48	21	74	-3.325	0.029
G199S	45	49	24	80	-3.674	0.021
G199S	46	51	24	6	6.68	0.003
G199S	104	131	38	28	3.412	0.027
G199S	200	248	17	41	-4.916	0.008
G199S	259	263	69	81	-3.538	0.024
R374S	3	6	20	65	-3.318	0.029
R374S	3	30	39	7	3.421	0.027
R374S	4	355	2	15	-4.836	0.008
R374S	7	349	32	18	3.239	0.032
R374S	7	355	64	41	2.83	0.047
R374S	8	24	30	65	-5.097	0.007
R374S	8	104	89	78	3.383	0.028
R374S	39	53	85	50	2.845	0.047
R374S	41	65	74	87	-3.406	0.027
R374S	43	51	47	7	3.417	0.027
R374S	44	51	27	2	3.829	0.019
R374S	45	53	0	14	-4.01	0.016
R374S	48	51	39	9	4.221	0.013
R374S	51	54	13	58	-5.033	0.007
R374S	51	82	8	51	-3.546	0.024
R374S	51	85	1	29	-9.405	0.001
R374S	51	86	6	51	-5.115	0.007
R374S	52	86	22	63	-2.852	0.046
R374S	56	83	35	13	2.895	0.044
R374S	100	104	39	24	3.798	0.019
R374S	100	125	19	7	4.532	0.011
R374S	104	131	38	23	3.052	0.038
R374S	106	348	49	75	-3.559	0.024
R374S	122	136	52	22	3.484	0.025
R374S	130	361	66	79	-4.319	0.012
R374S	135	372	56	0	2.985	0.041
R374S	135	374	61	0	3.567	0.023
R374S	135	377	44	93	-2.788	0.049
R374S	137	348	22	39	-4.015	0.016
R374S	137	376	31	0	3.302	0.03
R374S	141	376	18	0	3.437	0.026
R374S	145	170	11	23	-3.157	0.034
R374S	145	377	72	36	2.936	0.043
R374S	150	377	27	3	3.343	0.029
R374S	169	377	46	15	3.478	0.025
R374S	348	376	38	0	3.312	0.03
R374S	354	376	57	7	2.86	0.046
R374S	357	374	59	0	4.338	0.012
R374S	358	363	65	24	3.391	0.028

**R374S**	358	374	59	0	4.751	0.009
**R374S**	359	363	76	44	3.305	0.03
**R374S**	359	364	64	28	2.914	0.043
**R374S**	359	375	33	91	-3.972	0.017
**R374S**	360	374	16	0	3.604	0.023
**R374S**	363	374	79	2	11.273	0
**R374S**	370	374	42	91	-3.838	0.018
**R374S**	371	375	35	96	-4.043	0.016
**R374S**	372	375	43	100	-2.856	0.046
